# Increased AT_2_R expression is induced by AT_1_R autoantibody via two axes, Klf-5/IRF-1 and circErbB4/miR-29a-5p, to promote VSMC migration

**DOI:** 10.1038/s41419-020-2643-5

**Published:** 2020-06-08

**Authors:** Yan Sun, Yang Li, Meili Wang, Mingming Yue, Lina Bai, Jingwei Bian, Weiwei Hao, Jing Sun, Suli Zhang, Huirong Liu

**Affiliations:** 1 Department of Physiology & Pathophysiology, School of Basic Medical Sciences, Capital Medical University, 100069 Beijing, PR China; 2 Beijing Key Laboratory of Metabolic Disorder Related Cardiovascular Disease, Capital Medical University, 100069 Beijing, PR China; 3 Department of Physiology, Basic Medical College, Changzhi Medical College, 046000 Changzhi, Shanxi PR China

**Keywords:** Cell biology, Molecular biology

## Abstract

Vascular remodeling can be caused by angiotensin II type 1 receptor (AT_1_R) autoantibody (AT1-AA), although the related mechanism remains unknown. Angiotensin II type 2 receptor (AT_2_R) plays multiple roles in vascular remodeling through cross-talk with AT_1_R in the cytoplasm. Here, we aimed to explore the role and mechanism of AT_2_R in AT1-AA-induced vascular smooth muscle cell (VSMC) migration, which is a key event in vascular remodeling. In vitro and in vivo, we found that AT_2_R can promote VSMC migration in AT1-AA-induced vascular remodeling. Moreover, AT_2_R expression was upregulated via Klf-5/IRF-1-mediated transcriptional and circErbB4/miR-29a-5p-mediated posttranscriptional mechanisms in response to AT1-AA. Our data provide a molecular basis for AT1-AA-induced AT_2_R expression by transcription factors, namely, a circular RNA and a microRNA, and showed that AT_2_R participated in AT1-AA-induced VSMC migration during the development of vascular remodeling. AT_2_R may be a potential target for the treatment of AT1-AA-induced vascular diseases.

## Introduction

Vascular remodeling is closely related to various cardiovascular diseases, such as hypertension, atherosclerosis, and cardiomyopathy^1^. The migration of vascular smooth muscle cells (VSMCs) from arterial media to intima plays a key role in the development of vascular remodeling because of its contribution to arterial intima thickening and lumen stenosis^2,3^. The renin-angiotensin system (RAS) is one of the major mediators of vascular remodeling and related diseases by regulating VSMC migration, promoting inflammation, etc.^4^. In addition to angiotensin II (Ang II), Ang II type 1 receptor (AT_1_R) autoantibody (AT1-AA) is a newly discovered agonist of AT_1_R^5^, which is involved in pathological changes in vascular diseases^6–9^. Wallukat^10^ initially detected AT1-AA in the serum of pregnant women with preeclampsia. Ample evidence supports an important role for AT1-AA in vascular remodeling^11,12^, although the related molecular mechanism is not clear.

AT_1_R and Ang II type 2 receptor (AT_2_R), which are G-protein-coupled receptors, are the primary receptors of the RAS^13,14^. At present, most studies indicate that AT_1_R mediates Ang II-induced vascular injury, while AT_2_R protects against vascular injury^14^. Therefore, AT_2_R is a critical component of the “protective arm” of the RAS. However, there are also reports of AT_2_R mimicking the function of the AT_1_R receptor under some disease conditions^15^. For example, AT_2_R activation triggers tonic renal medullary vasoconstriction in renovascular hypertension^16^, and it also increases mesenchymal stem cell migration^17^. However, the roles of AT_2_R in changes of the VSMC migratory ability in vascular remodeling caused by AT1-AA and the underlying regulatory mechanism remain elusive. This study will provide insights into the multiple actions of AT_2_R in the cardiovascular system.

Recently, several studies have reported on the mechanism of AT_2_R regulation. Previous studies have found that interferon regulatory factor 1 (IRF-1) can regulate the expression of the AT_2_R gene^18–20^. However, the other transcription factors involved in the regulation of the AT_2_R gene are largely unknown. Klf-5 is a zinc finger-containing transcription factor^21^ that has a binding sequence in the region of the mouse AT_2_R promoter; however, whether Klf-5 regulates AT_2_R gene expression induced by AT1-AA is unclear. In addition to transcriptional regulation, posttranscriptional regulation plays an important role in gene expression. Circular RNAs (circRNAs) represent a novel class of noncoding RNAs (ncRNAs) generated by back splicing^22,23^. Accumulating evidence has shown that the microRNA (miRNA) sponge activity of circRNAs is a general phenomenon^24–26^. Nevertheless, whether and how circRNAs participate in the regulation of AT_2_R gene expression induced by AT1-AA are unknown.

In our study, we found that upregulation of AT_2_R expression promoted VSMC migration and participated in vascular remodeling induced by AT1-AA. AT_2_R was regulated through two pathways: Klf-5/IRF-1-mediated regulation at the transcriptional level and circErbB4/miR-29a-5p-mediated regulation at the posttranscriptional level. Our study suggested that AT1-AA promotes vascular remodeling via a distinct molecular mechanism relative to that of Ang II and indicated that more attention should be paid to AT_2_R in the treatment of AT1-AA-related vascular diseases.

## Methods

### Animals

All animal studies were approved by the Institutional Animal Care and Use Committee of the Capital Medical University (Beijing, China) and all efforts were made to minimize suffering. Eight-week-old male BALB/c mice were maintained in a 12:12 h light:dark cycle at an ambient temperature of 23−25 °C. After a 1-week acclimatization period, the animals were randomly assigned to the following four groups (*n* = 6−8 for each group): control, IgG, AT1-AA and AT1-AA + PD123319. The AT1-AA (50 μg/g) was injected into the mice via the tail vein, and an equal dose of normal saline or control IgG was injected into the mice in the control group and IgG group, respectively. The mice continued receiving injections that were the same as the first injection every 10 days over a month-long period. The AT1-AA + PD123319 group received the AT1-AA combined with the AT_2_R antagonist PD123319 (5 mg/kg/day), which was administered via intraperitoneal injection. Then thoracic aortas were isolated from the mice for analysis by Western blotting, PCR and immunofluorescence staining.

### Cell culture and treatment

Mouse aortic smooth muscle cells (MASMCs) and human embryonic kidney 293 A cells were maintained as previously reported^27^, and authenticated by short tandem repeat (STR) profiling and tested for mycoplasma contamination. Before stimulation and infection with plasmids, MASMCs were incubated in serum-free medium for 24 h. Ang II (A9525) and PD123319 (P186) were purchased from Sigma-Aldrich. AT1-AA, a monoclonal antibody against the human second extracellular loop of AT_1_R, was prepared via a specific method that has been previously described^28^, and it can imitate the biological activity of AT1-AA-positive patients.

### Microarray analysis

Circular RNA expression profiling was performed using an Arraystar Mouse circRNA Array V2 analysis (Arraystar, USA). Circular RNAs of MASMCs were extracted using QIAzol Lysis Reagent (QIAGEN, Catalog no. 79306) according to the manufacturer’s instructions. The sample preparation and microarray hybridization were performed based on the Arraystar’s standard protocols. Briefly, total RNAs were digested with Rnase R (Epicentre, Inc.) to remove linear RNAs and enrich circular RNAs. Then, the enriched circular RNAs were amplified and transcribed into fluorescent cRNA utilizing a random priming method (Arraystar Super RNA Labeling Kit; Arraystar). The labeled cRNAs were hybridized onto the Arraystar Mouse circRNA Array V2 (8 × 15 K, Arraystar). After washing slides, the arrays were scanned by the Agilent Scanner G2505C. Agilent Feature Extraction software (version 11.0.1.1) was used to analyze acquired array images.

### Morphometry and histology

Mice were euthanized, perfused and then fixed with 4% paraformaldehyde in 0.9% NaCl administered for 3 min through the left ventricle under physiological pressure. The thoracic aortic arteries were harvested, fixed with formalin and embedded in paraffin. Ten consecutive 4-μm-thick sections were prepared for haematoxylin and eosin staining. Images were acquired using a 3D Histech Pannoramic scanning system. Measurement of the medial thickness was performed in a blinded manner. For each section, four random, noncontiguous microscopic fields were examined.

### Noninvasive BP measurement

Blood pressure (BP) was measured with a tail-cuff noninvasive BP measurement system using volume pressure recording sensors (Softron, BP-98A, Japan). Mice were placed on a heated platform, and the BP values were the average of ten consecutive measurements.

### In vivo ultrasound studies

Noninvasive Doppler ultrasound imaging involved a Vevo2100 imaging system with an MS-550D (22−55 MHz) transducer (Visualsonics, Toronto, Canada). Measurements were taken in the supine position on a heated platform while the mice were anesthetized with 1.3% isoflurane with continuous electrocardiogram monitoring, and the mouse heart rates ranged from 325 to 375 beats per minute. The pulse wave velocity (PWV) was calculated as the ratio of the distance and time delay of the systolic pulse wave between the left subclavian artery and renal artery. The average diameter of the thoracic aorta was analyzed and calculated from transverse M-mode ultrasonography.

### Cell proliferation assay

MASMC proliferation assays were performed with the BrdU Cell Proliferation Assay kit (Millipore) according to the manufacturer’s recommendations. Cells were labeled for 6 h, and OD readings were performed at 450 nm. All groups were evaluated in a minimum of three separate wells per experiment.

### Wound-healing assay

Cells were seeded with the same numbers in six-well plates with different stimulations. When the cells grew to 95% confluence, scratch wounds were created using 100-μl sterile pipette tips. To remove the suspended cells, the plates were washed with phosphate buffered saline (PBS) twice. Images were captured in three defined fields at 0 and 24 h, respectively.

### Transwell migration assay

MASMCs were seeded in 24-well Boyden chambers with 8-μm pores (Corning, NY, USA) and subjected to cell migration assays. The lower chamber was filled with 600 μl of Dulbecco’s modified Eagle’s medium (DMEM), and MASMCs cells with different stimulations were placed in the upper chamber. After culturing for 12 h, the cells from the upper chambers of the transwells were removed, and the migrated cells on the undersides of the membranes were fixed. After fixation, the cells were stained with crystal violet and counted on the lower side of the membrane using ImageJ software.

### Immunostaining

Immunofluorescence staining was performed with 4-μm paraffin cross-sections from the thoracic aorta of mouse. After deparaffinization with xylene and rehydration, the slides were preincubated with 10% normal goat serum and then incubated with the primary antibodies anti-MMP-2 (ab92536, Abcam) and anti-MMP-9 (ab38898, Abcam). MASMCs were fixed in 4% paraformaldehyde for 5 min at room temperature and then were washed with PBS, followed by incubation in 10% normal goat serum blocking solution for 30 min in a humidified chamber at room temperature. The cells were incubated in anti-Klf-5 (GTX103289, GeneTex) and anti-IRF-1 (sc-514544, Santa Cruz) for 2 h at room temperature, washed with PBS, and incubated in fluorescein-conjugated secondary antibodies for 60 min. In each experiment, 4′,6-diamidino-2-phenylindole (DAPI) (157574, MB biomedical) was used for nuclear counterstaining. Images were captured by confocal microscopy (DM6000 CFS, Leica) and processed with LAS AF software.

### Phalloidin staining for actin stress fibers

MASMCs were fixed in 4% paraformaldehyde and permeabilized with 0.1% Triton X-100 at room temperature for 10 min, followed by tetramethyl rhodamine isothiocyanate (TRITC)-phalloidin (Sigma) staining for 30 min in the dark. Staining with DAPI was performed to visualize nuclear localization. Confocal microscopy was performed with a Confocal Laser Scanning Microscope System (Leica).

### Western blot analysis

Protein extraction and quantification were performed as previously reported^29^. The following antibodies were used: anti-AT_1_R (1:1000, GTX89149, GeneTex), anti-AT_2_R (1:1000, ab92445, Abcam), anti-Klf-5 (1:500, GTX103289, GeneTex), anti-IRF-1 (1:1000, ab186384, Abcam), anti-QKI (1:1000, ab126742, Abcam), anti-ADAR1(1:500, 14330-1-AP, Proteintech) and anti-β-actin (1:1000, sc-47778, Santa Cruz). All experiments were repeated three times.

### CoIP assay

CoIP was performed as previously described^30^. In brief, the cell lysates were immunoprecipitated with anti-IRF-1 (sc-514544; Santa Cruz) and anti-Klf-5 (GTX103289; Gene Tex) respectively for 1 h at 4 °C, then incubated with protein A-agarose overnight at 4 °C. Protein A-agarose-antigen-antibody complexes were collected by centrifugation at 12,000 × *g* for 2 min at 4 °C, and washed five times with 1 ml immunoprecipitation-HAT buffer (50 mM Tris-HCl, pH 8.0, 150 mM NaCl, 5 mM ethylenediamine tetraacetic acid (EDTA), 0.5% NP-40, and 0.1 mM Phenylmethylsulfonyl Fluoride (PMSF)) for 20 min each time at 4 °C. The bound proteins were resolved using SDS-PAGE, followed by Western blotting with anti-Klf-5 and anti-IRF-1 antibodies.

### Isolation of RNA and PCR

MASMCs and thoracic aortas were lysed by using QIAzol Lysis Reagent (QIAGEN, Catalog no. 79306). Supplementary Table 1 lists the primer sequences. Other sequences of circRNA primers will be provided as required.

### RNase R treatment

RNase R treatment was carried out according to the manufacturer’s instructions. Briefly, 5 μg of total RNA was incubated for 20 min at 37 °C with or without 20 U/μl RNase R (Epicentre Technologies, Madison, WI), and the resulting RNA was purified using the RNeasy MinElute cleaning Kit (QIAGEN).

### Biotinylated-oligonucleotide pulldown of RNA

To detect the circErbB4 and miR-29a-5p interaction, biotin pulldown was carried out as previously described^27^. In brief, MASMCs were cross-linked with 1% formaldehyde in PBS for 10 min at room temperature, then quenched with 0.125 M glycine for 5 min. The cells were resuspended in lysis buffer (50 mM Tris, pH 7.0, 10 mM EDTA, and 1% sodium dodecyl sulfate (SDS); with freshly added 1 mM dithiothreitol (DTT), complete protease inhibitor, and 0.1 U/μl RNase inhibitor) on ice for 10 min and were sonicated. The cell lysate was diluted in two times volume with hybridization buffer (750 mM NaCl, 1% SDS, 50 mM Tris, pH 7.0, 1 mM EDTA, 15% formamide, 1 mM DTT, protease inhibitor, and 0.1 U/μl RNase inhibitor). 100 pmol biotin probes were added. Streptavidin Dynabeads (Life Technologies) were blocked for 2 h at 4 °C in lysis buffer containing 1 mg/ml yeast tRNA and 1 mg/ml bovine serum albumin (BSA) and washed twice with 1 ml lysis buffer. One hundred microliters washed/blocked Dynabeads was added per 100 pmol of biotin probes, and the whole mix was then rotated for 30 min at 37 °C. Beads were captured by magnets (Life Technologies) and washed five times with wash buffer (2× Saline Sodium Citrate (SSC), 0.5% SDS, and 0.1 mM DTT and PMSF). Beads were then subjected to RNA elution with buffer (Tris 7.0, 1% SDS).

### FISH

For circRNA fluorescence in situ hybridization (FISH), cells were fixed in 4% paraformaldehyde for 5 min at room temperature, permeabilized with 0.5% Triton X-100 and washed with PBS. The process was performed using the Ribo^TM^ Fluorescent In Situ Hybridization Kit (RiboBio, China).

For miRNA FISH, cultured cells were prepared as described previously^31^. miRNA FISH was conducted with the miRCURY LNA^TM^ microRNA ISH Optimization Kit (90001, QIAGEN, Germany) and a miR-29a-5p double-fluorescein (both the 5′ and the 3′ ends were labeled with FITC) FISH probe (Genepharma, China).

### ChIP assay

A ChIP assay was performed as described previously^30,31^. The CHIP assay was carried out according to the manufacturer’s instructions for ChIP KIT (17-371, Millipore). In brief, MASMCs were treated with 1% formaldehyde for 10 min to cross link proteins with DNA. The cross-linked chromatin was then prepared and sonicated to an average size of 400–600 bp. The samples were diluted tenfold and then precleared with protein A-agarose/salmon sperm DNA for 30 min at 4 °C. The DNA fragments were immunoprecipitated overnight at 4 °C with the anti-Klf-5, or anti-IRF-1 antibodies. After cross-linking reversal, Klf-5 or IRF-1 occupancy on the AT_2_R gene intron was examined. All results were determined by qRT-PCR. The ChIP primer sequences are provided in Supplementary Table 1. All results were determined by quantitative qRT-PCR. Each experiment was replicated at least three times.

### Luciferase assay

Human embryonic kidney 293A cells were maintained as previously described^32^. For the luciferase assays, 293A cells were transfected with luciferase reporter plasmids using Lipofectamine 2000 (Invitrogen) according to the manufacturer’s protocol. The cells were harvested, and luciferase activity was measured using the Dual-Glo Luciferase Assay Kit (Promega) after transfection. The specific target activity is expressed as the relative activity ratio of firefly luciferase to Renilla luciferase. All constructs were evaluated in a minimum of three separate wells per experiment.

### Transfection of siRNAs, plasmids, miRNAs

Small interfering RNAs (siRNAs) targeting mouse circErbB4 (si-circErbB4) was designed and synthesized by RiboBio (Guangzhou, China). The siRNA sequence was as follows: circErbB4 siRNA (si-circErbB4), 5′-GAGCTGAGAATTGTATCTA-3′. Nonspecific siRNA (si-Control), siRNA specific for mouse AT_2_R siRNA (si-AT_2_R), IRF-1 siRNA (si-IRF-1), Klf-5 siRNA (si-Klf-5), QKI siRNA (si-QKI) and ADAR1 siRNA (si-ADAR1) were purchased from Santa Cruz Biotechnology. The plasmid of circErbB4 (pLVX-circErbB4) was generated via Likely Biotechnology, Beijing. The expression plasmids of AT_2_R, IRF-1 and Klf-5 were created by the placement of mouse AT_2_R, IRF-1 and Klf-5 cDNA into the pcDNA3.1 vector. The sequences of the AT_2_R gene promoter containing the Klf-5 and IRF-1 binding sites or their mutant sequences were inserted into the pGL3-Basic vector. Sequences of the circErbB4, AT_2_R and QKI genes’ 3′UTR containing the miR-29a-5p target site or its mutant sequences were inserted into the pmirGLO Dual-Luciferase miRNA Target Expression Vector. miR-29a-5p-mimic, inhibitor and control RNAs were designed and synthesized by Gene Pharma (Shanghai, China). Transfection was performed using Lipofectamine 2000 following the manufacturer’s instructions. Twenty hours following transfection, MASMCs were treated with AT1-AA, miR-29a-5p-mimic, or anti-miR-29a-5p and then harvested and lysed for Western blot, PCR and luciferase assays.

### Statistical analysis

All the data are presented as the mean ± SD. All the data were normally distributed. Differences between two groups were assessed using analysis of variance followed by a Student’s *t* test. A value of *P* < 0.05 was considered statistically significant. Sample size was chosen according to previous observations, which performed similar experiments to observe significant results. Statistical analysis was performed using GraphPad Prism 8 software (GraphPad Software, San Diego, CA, USA).

## Results

### AT1-AA induces VSMC migration via cytoskeletal reorganization but not proliferation

**Fig. 1:**
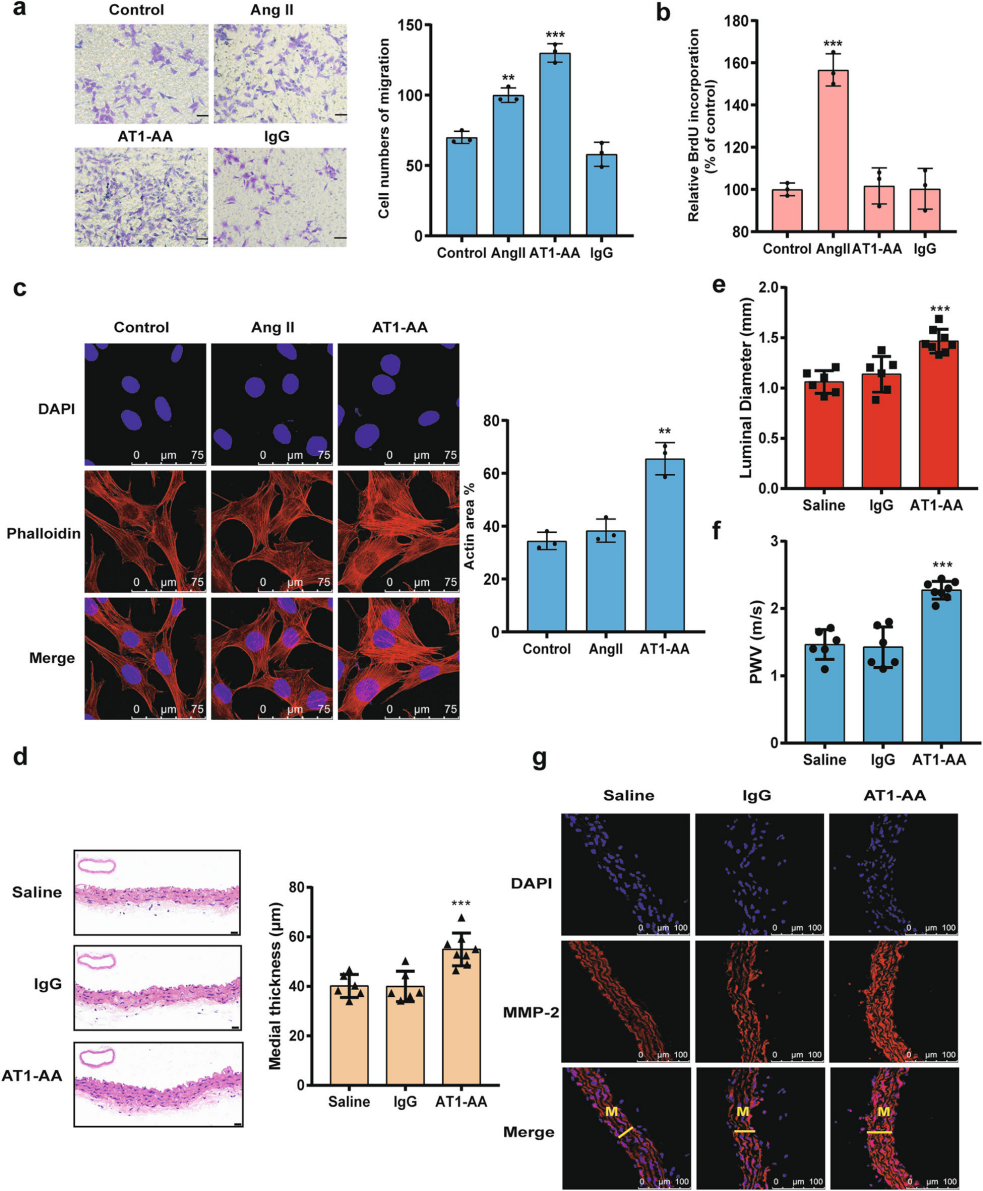
AT1-AA induces cell migration in vitro and in vivo. **a** Migration changes were detected by a transwell chamber migration assay. The changes were observed at 0 and 24 h. The stimulation doses were 10^−7^ M. Scale bars = 100 μm. Data are presented as mean ± SD (**p < 0.01; ***p < 0.001; *n* = 3). **b** A BrdU assay was performed to assess cell proliferation (***p < 0.001; *n* = 3). **c** MASMCs were fluorescently stained for F-actin with TRITC-phalloidin, and imaged with a laser scanning confocal microscope. The right panel shows actin area analyses from three independent experiments (**p < 0.01). **d** Arterial sections from normal saline-, IgG- and AT1-AA-treated mice were stained with hematoxylin and eosin (HE). The right panel shows the quantitative analysis of the medial thickness of the aortas from the saline-, IgG- and AT1-AA-treated mice. Scale bars = 20 μm (***p < 0.001; *n* = 6−8). **e** The luminal diameter in the thoracic aorta region was quantified by transverse M-mode ultrasonography (***p < 0.001; *n* = 6−8). **f** Pulse wave velocity (PWV) measurements were taken after 1 month in AT1-AA-treated, IgG-treated and saline-treated mice (***p < 0.001; *n* = 6−8). **g** Fluorescent staining of aortic section with an anti-MMP-2 antibody and DAPI was amplified.

To determine the effect of AT1-AA on VSMC migration, MASMCs were treated with AT1-AA, Ang II or IgG in scratch wound assays and transwell assays. Unexpectedly, the cells treated with AT1-AA showed a stronger migratory ability than those treated with Ang II, while IgG had no effect on cell migration (Fig. 1a and Supplementary Fig. S1a). However, a BrdU assay showed that Ang II induced cell proliferation while AT1-AA did not (Fig. 1b), suggesting that the AT1-AA-induced increase in MASMC migration was not mediated by proliferation. Knowing that F-actin cytoskeletal networks can regulate cellular shape changes and force cell migration, we observed the actin structure by staining cells with rhodamine phalloidin, which is a probe for filamentous actin, after treatment with AT1-AA or Ang II. The results showed that actin filaments were recruited into thick, long actin bundles in the AT1-AA-treated cells, while no such phenomenon was observed in the Ang II treatment group (Fig. 1c). These results suggested that unlike the migration caused by Ang II through proliferation, the increased migration induced by AT1-AA occurred via cytoskeletal reorganization.

To further clarify the effect of AT1-AA in VSMC migration in vivo, an AT1-AA-positive mouse model was established by repeatedly injecting AT1-AA via the tail vein over 4 weeks. Compared with the saline- or IgG-treated mice, the marked medial expansion and increased mean aortic diameter were observed in the thoracic aorta of the AT1-AA-treated mice (Fig. 1d, e), as well as aortic pulse wave velocity (PWV) (Fig. 1f). Furthermore, matrix metalloproteinases (MMPs) degrade the basement membrane and the extracellular matrix, facilitating VSMC migration. As shown in Fig. 1g and Supplementary Fig. S1b, AT1-AA infusion resulted in marked increases in MMP-2 and MMP-9 expression in the thoracic aorta media of the mice. These findings indicated that AT1-AA can promote VSMC migration during the development of vascular remodeling.

### AT1-AA participates in VSMC migration by increasing AT2R gene expression

**Fig. 2:**
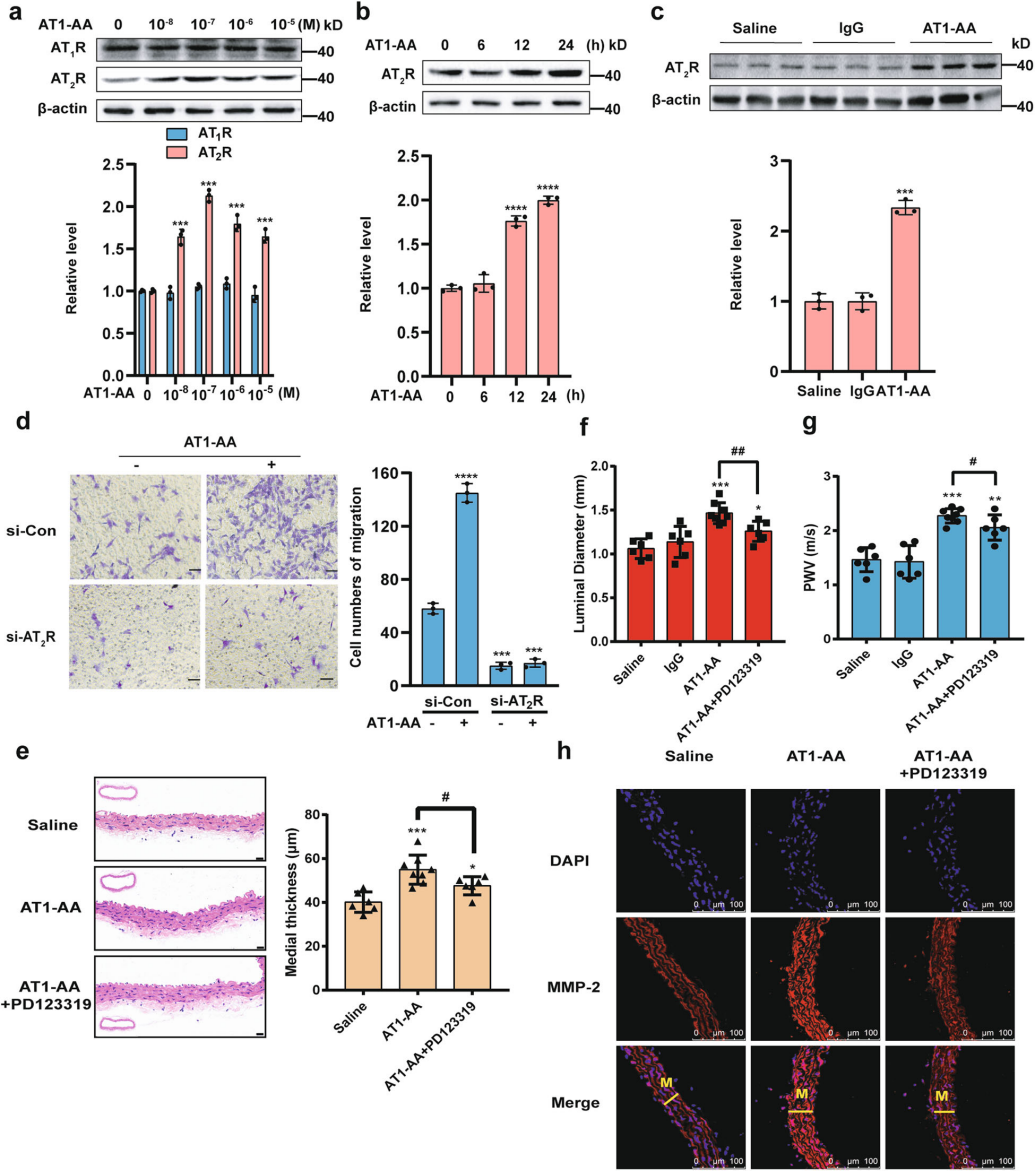
AT1-AA promotes the expression of AT_2_R which participates in migration. **a** AT_1_R and AT_2_R protein levels were analyzed by Western blotting. The down panel shows densitometric analyses from three independent experiments. Data are presented as mean ± SD (***p < 0.001). **b** Western blotting was used to assess AT_2_R expression (****p < 0.0001; *n* = 3). **c** The AT_2_R protein levels in aortic arteries from mice treated with saline, IgG or AT1-AA were determined by Western blotting (***p < 0.001; *n* = 3). **d** MASMCs were transfected with si-Con or si-AT_2_R, treated with or without the AT1-AA and stained in a migration assay. Scale bars = 100 μm (****p < 0.0001; ***p < 0.001; *n* = 3). **e** HE staining of aortic walls was performed. AT1-AA + PD123319 group was compared with saline and AT1-AA groups in Fig. 1d. Scale bars = 20 μm (*p < 0.05; ***p < 0.001 vs. saline; ^#^*p* < 0.05; *n* = 6−8). **f** The luminal diameter of the thoracic aorta was quantified in saline-treated mice, IgG-treated mice, and AT1-AA (with or without PD123319)-treated mice (*p < 0.05, ***p < 0.001 vs. saline; ^##^*p* < 0.01; *n* = 6−8). **g** PWV was measured after 1 month in mice treated with different stimuli (**p < 0.01, ***p < 0.001 vs. saline; ^#^*p* < 0.05; *n* = 6−8). **h** The localization of MMP-2 was examined by fluorescent staining. AT1-AA + PD123319 group was compared with saline and AT1-AA groups in Fig. 1g.

Considering that AT_2_R participates in migration^17,33^, we determined whether AT_2_R is involved in the changes in MASMCs caused by AT1-AA. As shown in Fig. 2a, the AT1-AA increased AT_2_R protein expression in a dose-dependent manner for 24 h, but did not affect the AT_1_R protein level. An opposite result was obtained in the Ang II treatment group (Supplementary Fig. S1c), and IgG did not affect either protein (Supplementary Fig. S1d). Then, we chose a concentration of 10^−7^ M to demonstrate that AT1-AA significantly promoted the expression of AT_2_R in a time-dependent manner (Fig. 2b). Consistent with these results, the Western blot analysis results also showed increased expression of AT_2_R in the thoracic aorta of mice injected with AT1-AA compared to that in the thoracic aorta of mice injected with saline or IgG (Fig. 2c). Because AT1-AA activates the downstream signaling pathway mainly through AT_1_R^5^, we blocked AT_1_R and found that AT1-AA did not increase the AT_2_R protein level after the si-AT_1_R treatment (Supplementary Fig. S1e), indicating the critical role of AT_1_R in AT1-AA-induced high expression of AT_2_R.

To verify whether AT_2_R participates in AT1-AA-induced migration in vitro and in vivo, MASMCs were transfected with si-AT_2_R (Supplementary Fig. S2a, b), and the effect of AT1-AA on MASMC migration was completely blocked (Fig. 2d and Supplementary Fig. S2c). Because the nonpeptide-specific AT_2_R antagonist PD123319 effectively prevents AT_2_R signaling in an aortic disease mouse model, mice were treated with both AT1-AA and PD123319. The results showed that PD123319 attenuated the harmful effects of AT1-AA on medial thickness, aortic dilation, PWV and MMP-2 and MMP-9 expression (Fig. 2e–h and Supplementary Fig. S2d); however, no alteration in BP was observed when comparing the PD123319-treated mice to the AT1-AA-treated mice (Supplementary Fig. S2e). These findings suggest that AT_2_R activation plays important roles in migration induced by AT1-AA.

### Klf-5 and IRF-1 mediate AT1-AA-induced expression of the AT2R gene

**Fig. 3:**
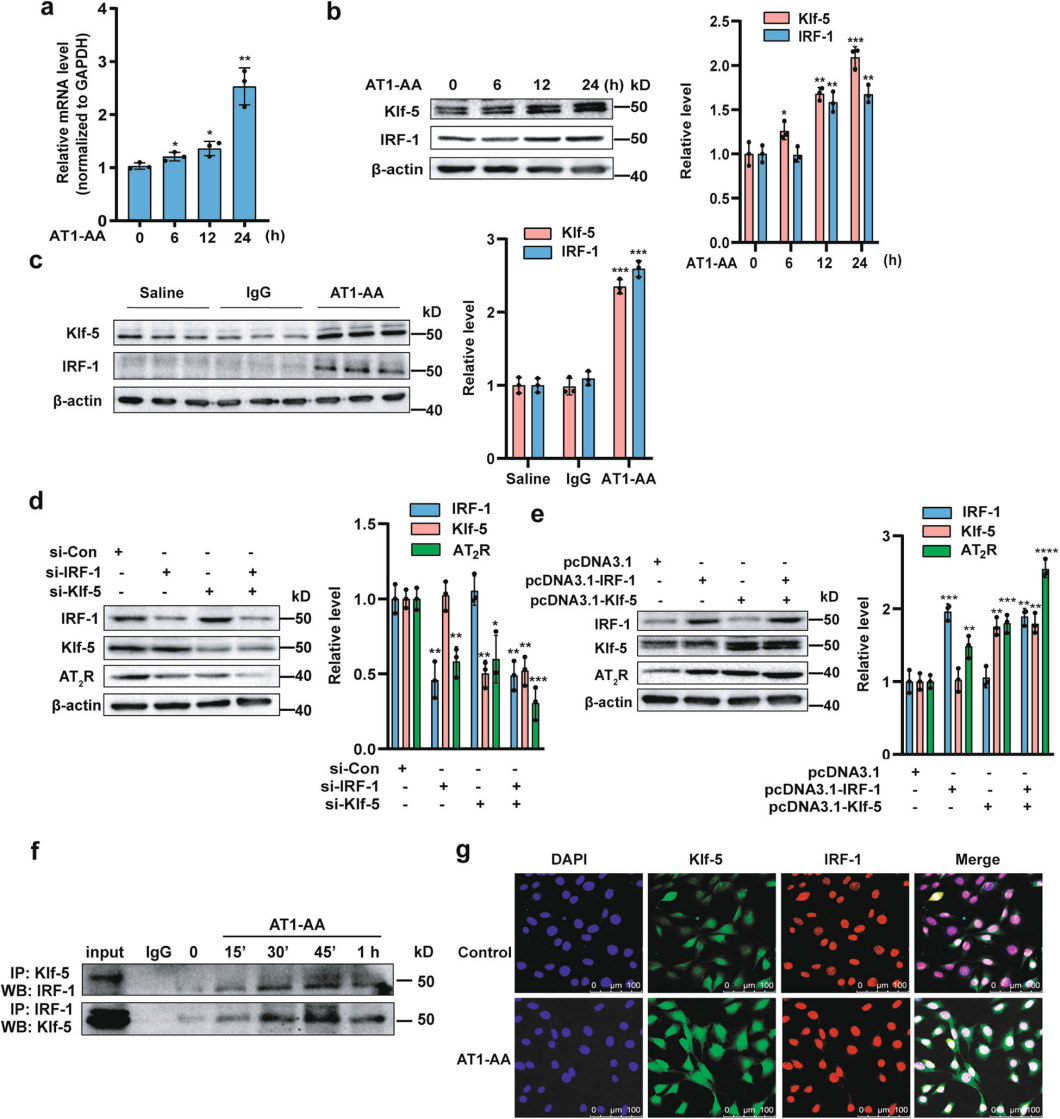
Klf-5 and IRF-1 cooperatively enhance the expression of AT_2_R. **a** The relative mRNA level of AT_2_R was examined by qRT-PCR and is presented after normalizing to the GAPDH level. Data are presented as mean ± SD (*p < 0.05; **p < 0.01; *n* = 3). **b** Western blotting was used to detect Klf-5 and IRF-1 protein levels (*n* = 3). The right panel shows densitometric analyses from three independent experiments (*p < 0.05; **p < 0.01; ***p < 0.001). **c** The protein levels of Klf-5 and IRF-1 in arteries from the saline, IgG and AT1-AA groups were examined by Western blotting (***p < 0.001; *n* = 3). **d** MASMCs were transfected with si-IRF-1, si-Klf-5, or both. IRF-1, Klf-5 and AT_2_R protein levels were analyzed by Western blotting (*p < 0.05; **p < 0.01; ***p < 0.001; *n* = 3). **e** Western blotting was used to detect IRF-1, Klf-5 and AT_2_R levels in MASMCs transfected with pcDNA3.1-IRF-1, pcDNA3.1-Klf-5, or both (**p < 0.01; ***p < 0.001; ****p < 0.0001; *n* = 3). **f** The interaction between Klf-5 and IRF-1 was examined by reciprocal coimmunoprecipitation (CoIP). **g** The locations of Klf-5 and IRF-1 were examined by fluorescent staining.

To detect the mechanism by which AT1-AA upregulates AT_2_R expression, we confirmed that AT1-AA increased AT_2_R but not AT_1_R mRNA levels in a dose- and time-dependent manner (Fig. 3a and Supplementary Fig. S3a). We wanted to confirm whether AT_2_R expression is regulated by Klf-5 or IRF-1 in MASMCs and found that AT1-AA increased Klf-5 and IRF-1 protein levels (Fig. 3b). In vivo, Klf-5 and IRF-1 levels were obviously increased in AT1-AA-positive mice (Fig. 3c). To gain further insights into the roles of Klf-5 and IRF-1 in AT_2_R expression, IRF-1 or Klf-5 was silenced, which decreased AT_2_R expression, and coinfection with IRF-1- and Klf-5-specific small interfering RNAs (siRNAs) almost completely inhibited AT_2_R expression (Fig. 3d). In contrast, overexpression of these molecules enhanced AT_2_R expression, which could be further increased when the molecules were used in combination (Fig. 3e). A coimmunoprecipitation (CoIP) experiment revealed that Klf-5 was associated with IRF-1 and AT1-AA increased their interactions, which peaked at 45 min and subsequently decreased, although not to control levels (Fig. 3f). As shown in Fig. 3g, after exposure to AT1-AA, the overlapping distributions of Klf-5 and IRF-1 were increased in the nuclear region of MASMCs. Taken together, our studies demonstrated that Klf-5 and IRF-1 regulated the expression of AT_2_R induced by AT1-AA.

### Klf-5 and IRF-1 cooperatively promote the transcription of AT2R under AT1-AA treatment

**Fig. 4:**
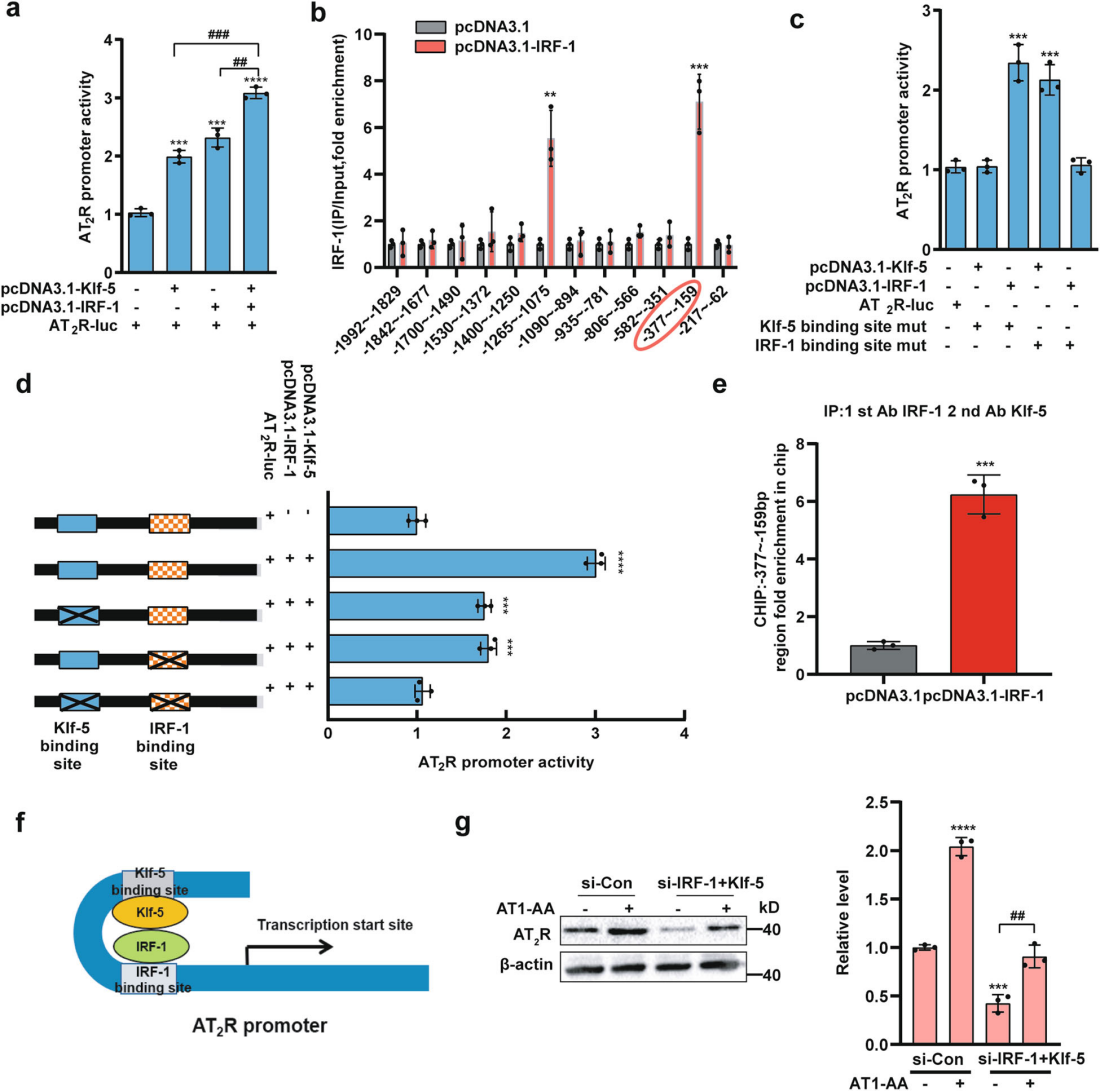
Klf-5 and IRF-1 regulate the transcription of the AT_2_R gene. **a** A luciferase reporter controlled by the AT_2_R promoter was transfected into HEK 293A cells with plasmids expressing Klf-5, IRF-1 or both. Data are presented as mean ± SD (***p < 0.001; ****p < 0.0001; ^##^*p* < 0.01; ^###^*p* < 0.001; *n* = 3). **b** ChIP analysis was used to analyze IRF-1 occupancy at the AT_2_R promoter (**p < 0.01; ***p < 0.001; *n* = 3). **c** HEK 293A cells were transfected with wild-type AT_2_R promoter-reporter constructs or constructs with mutated Klf-5 and IRF-1 binding sites with pcDNA3.1-Klf-5 and pcDNA3.1-IRF-1 (***p < 0.001; *n* = 3). **d** The mutations in the binding sites of Klf-5 and IRF-1 on the AT_2_R promoter region are represented in the left part of the panel (***p < 0.001; ****p < 0.0001; *n* = 3). **e** A two-step ChIP assay identified the interaction between Klf-5 and IRF-1 (***p < 0.001; *n* = 3). **f** A model of the transcriptional complex formed by Klf-5 and IRF-1 at the AT_2_R promoter is shown. **g** MASMCs were infected with si-Con or si-Klf-5+si-IRF-1 for 24 h and then treated with or without AT1-AA for an additional 12 h, and the AT_2_R expression level was detected by Western blot analysis (***p < 0.001; ****p < 0.0001; ^##^*p* < 0.01; *n* = 3).

The above results clearly suggested that Klf-5 and IRF-1 cooperatively enhanced AT_2_R expression. Similarly, cotransfection of Klf-5 and IRF-1 enhanced AT_2_R promoter activity (Fig. 4a). To detect the occupancies of Klf-5 and IRF-1 at the AT_2_R promoter, 12 pairs of primers that covered a 2.0-kb range of the AT_2_R promoter region were used (Supplementary Fig. S3b). Chromatin immunoprecipitation (ChIP) analysis showed that IRF-1 and Klf-5 were highly concentrated in the regions from −377 to −159 bp and −1265 to −1075 bp in the AT_2_R promoter, and these two positions contained IRF-1 and Klf-5 binding sites, respectively (Fig. 4b and Supplementary Fig. S3c). As shown in Fig. 4c, mutation of the Klf-5 binding site removed the promotive effect of Klf-5 but not that of IRF-1. Similarly, mutation of the IRF-1 binding site did not affect Klf-5 function. When these sites were mutated simultaneously, the AT_2_R promoter activity was not different from that of the wild-type promoter (Fig. 4d). To further identify the interaction between Klf-5 and IRF-1, a two-step ChIP assay was performed (Fig. 4e and Supplementary Fig. S3d). These results suggested that Klf-5 formed a stable complex with IRF-1 and these proteins bound to their binding sites in the AT_2_R promoter (Fig. 4f). Furthermore, we found that AT1-AA partly reversed the inhibitory effect of si-IRF-1+si-Klf-5 on the AT_2_R protein level (Fig. 4g, lane 4 versus lane 3). Unexpectedly, the AT1-AA did not reverse this phenomenon at the mRNA level (Supplementary Fig. S3e, lane 4 versus lane 3). These results explained that AT1-AA regulated AT_2_R expression not only at the transcriptional level but also at the posttranscriptional level via miRNAs or circRNAs.

### AT1-AA induces circErbB4 formation that upregulates AT2R expression

**Fig. 5:**
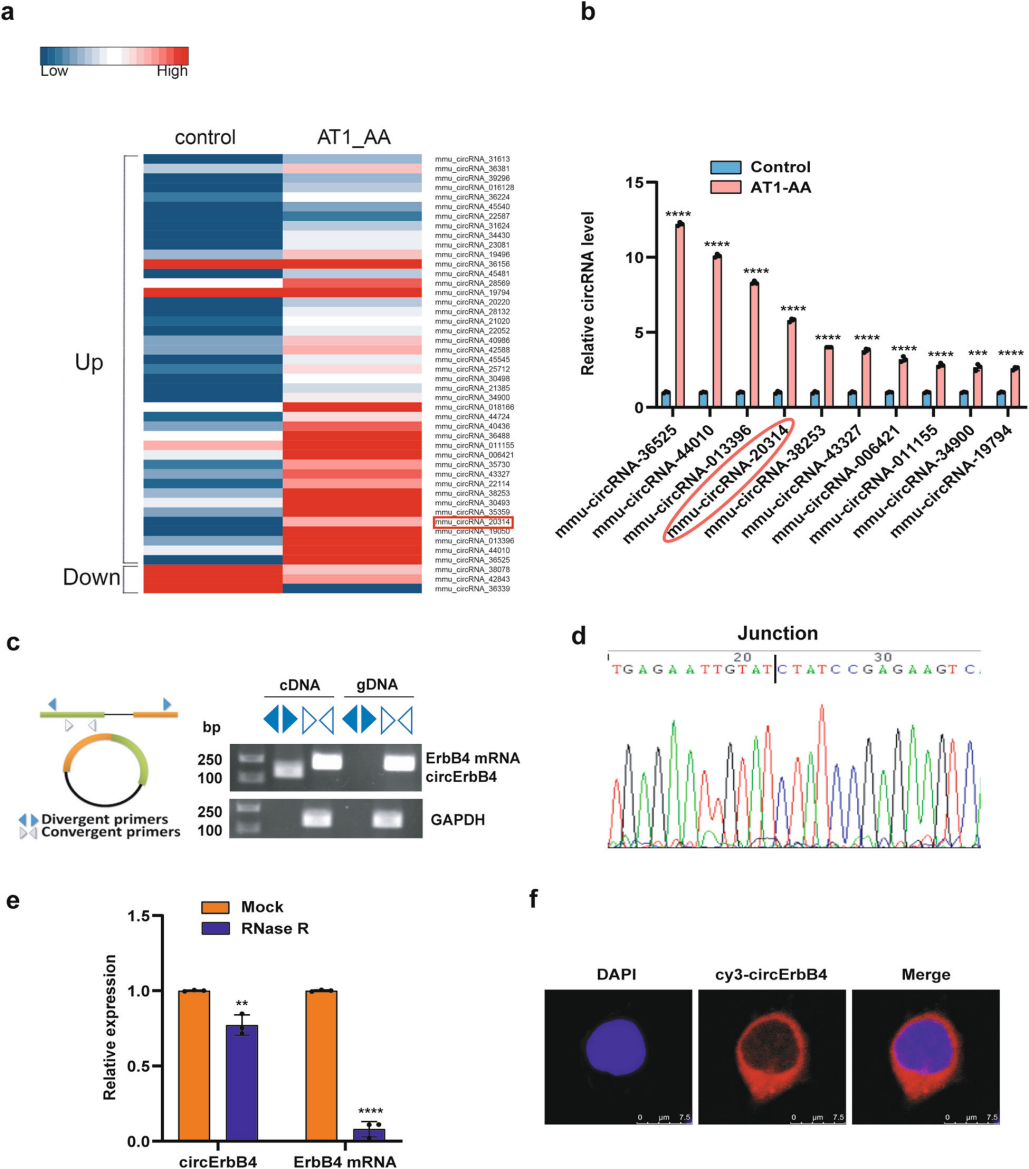
AT1-AA induces circErbB4 expression in MASMCs. **a** Hierarchical clustering analysis identified differentially expressed circRNAs (over a 2.0-fold change). **b** qRT-PCR was used to validate the expression of ten significantly upregulated circRNAs. Data are presented as mean ± SD (****p < 0.0001; ***p < 0.001; *n* = 3). **c** RT-PCR was used to verify that circErbB4 is a circRNA. **d** Sanger sequencing confirmed the head-to-tail junction of circErbB4. **e** qRT-PCR was performed to evaluate circErbB4 and ErbB4 mRNA in MASMCs treated with RNase R (**p < 0.01; ****p < 0.0001; *n* = 3). **f** RNA fluorescence in situ hybridization for circErbB4 was performed.

To determine whether circRNAs are involved in the AT1-AA-induced posttranscriptional regulation of AT_2_R, an Arraystar mouse circRNA microarray was performed with MASMCs. A heat map of 47 circRNAs shows that compared with the control group, 44 circRNAs exhibited upregulated expression and 3 exhibited downregulated expression (>2-fold) in the AT1-AA group (Fig. 5a). Then, we selected ten upregulated circRNAs according to their raw microarray signals and fold changes. The results showed that the expression of mmu-circRNA-20314, whose parental gene is ErbB4, was significantly upregulated in the AT1-AA group (Fig. 5b). We used convergent and divergent primers to amplify total RNA and circular RNA transcripts derived from the ErbB4 gene, respectively, by RT-PCR (Fig. 5c), and the PCR products were confirmed by DNA sequencing (Fig. 5d). RNase R digestion dramatically reduced the ErbB4 mRNA level, but it had a smaller effect on the circErbB4 level (Fig. 5e). The FISH results showed that circErbB4 was mostly located in the cytoplasm in MASMCs (Fig. 5f).

**Fig. 6:**
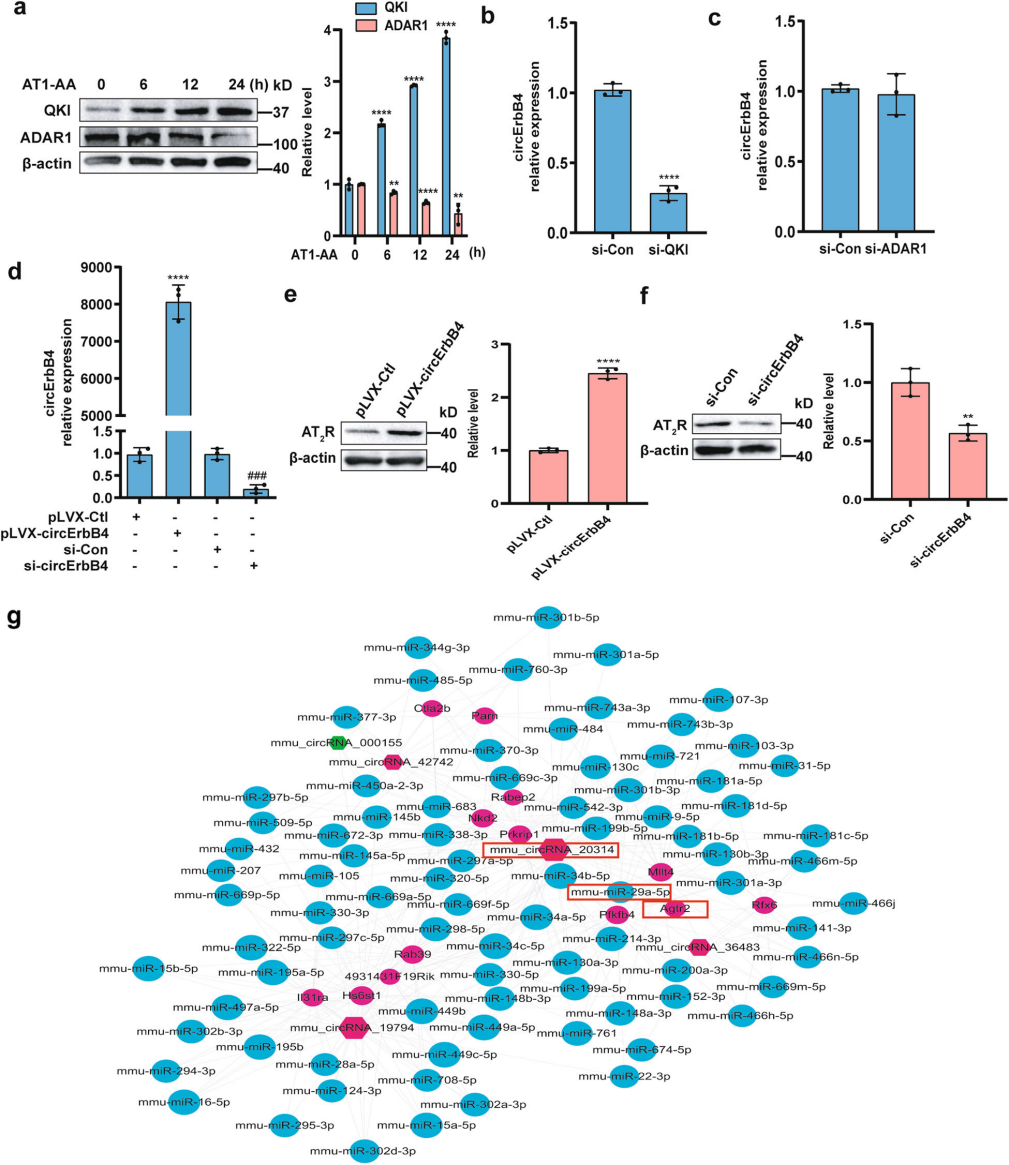
QKI participates in the expression of circErbB4, which can upregulate AT_2_R levels. **a** The expression levels of QKI and ADAR1 were measured by Western blotting. Data are presented as mean ± SD (**p < 0.01; ****p < 0.0001; *n* = 3). **b**, **c** MASMCs were transfected with si-QKI or si-ADAR1, and qRT-PCR was used to detect circErbB4 expression (****p < 0.0001; *n* = 3). **d** MASMCs were transfected with pLVX-circErbB4, si-circErbB4 or the corresponding control. circErbB4 expression was analyzed by qRT-PCR (****p < 0.0001 vs. pLVX-Ctl; ^###^*p* < 0.001 vs. si-Con; *n* = 3). **e**, **f** The expression level of AT_2_R in MASMCs transfected with pLVX-circErbB4 or si-circErbB4 was assessed by Western blotting (**p < 0.01; ****p < 0.0001; *n* = 3). **g** The red and green nodes represent upregulated and downregulated circRNAs, and the blue and purple nodes represent miRNAs and mRNAs, respectively. The enlarged red marker shows circRNA-20314-miR-29a-5p-Agtr2 interactions.

While investigating whether the RNA-binding protein Quaking (QKI)^34^ or RNA-editing enzyme ADAR1^35,36^ is responsible for AT1-AA-induced circErbB4 formation, we found that AT1-AA increased QKI expression and decreased ADAR1 expression (Fig. 6a). Notably, knocking down QKI expression markedly decreased circErbB4 formation (Fig. 6b and Supplementary Fig. S4a), whereas knocking down ADAR1 expression did not have a significant effect on circErbB4 expression (Fig. 6c and Supplementary Fig. S4b). Thus, we concluded that QKI participated in AT1-AA-induced circErbB4 formation. Given that AT1-AA increased AT_2_R expression and circErbB4 formation, we sought to determine the relationship between AT_2_R and circErbB4 by overexpressing or knocking down circErbB4 (Fig. 6d). CircErbB4 overexpression or knockdown increased or decreased the AT_2_R protein level, respectively (Fig. 6e, f). Knowing that circRNAs can function as miRNA sponges^37^, a circRNA-miRNA-mRNA network was constructed using Cytoscape (Fig. 6g).

### CircErbB4 upregulates AT2R expression by acting as a miR-29a-5p sponge, which suppresses AT2R expression by targeting the AT2R 3′-UTR in MASMCs

**Fig. 7:**
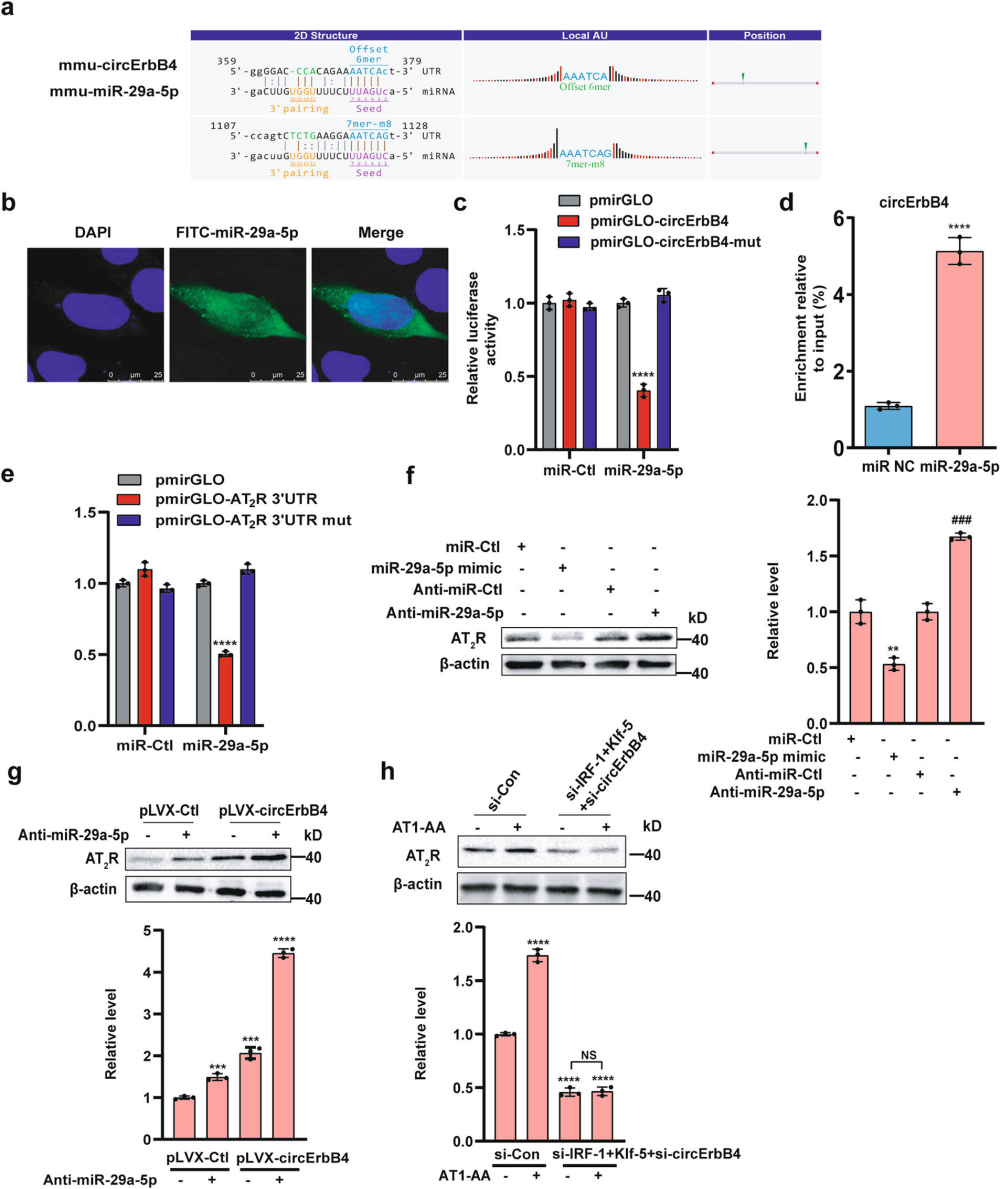
circErbB4 upregulates AT_2_R expression by acting as a miR-29a-5p sponge. **a** The miR-29a-5p-binding site in the circErbB4 sequence was predicted. **b** The location of miR-29a-5p in MASMCs was assessed. **c** HEK 293 A cells were cotransfected with pmirGLO-circErbB4 or pmirGLO-circErbB4 mut and an miR-29a-5p-mimic or miR-Ctl. Data are presented as mean ± SD (****p < 0.0001 vs. pmirGLO or pmirGLO-circErbB4 mut; *n* = 3). **d** qRT-PCR was used to detect circErbB4 enriched from MASMC lysates with a biotinylated-oligonucleotide probe for miR-29a-5p (****p < 0.0001; *n* = 3). **e** HEK 293A cells were cotransfected with the miR-29a-5p-mimic and pmirGLO-AT_2_R 3′-UTR or pmirGLO-AT_2_R 3′-UTR mut (****p < 0.0001 vs. pmirGLO or pmirGLO-AT_2_R 3′-UTR mut; *n* = 3). **f** MASMCs were transfected with the miR-29a-5p-mimic or anti-miR-29a-5p, and AT_2_R expression was analyzed by Western blotting (**p < 0.01 vs. miR-Ctl; ^###^*p* < 0.001 vs. Anti-miR-Ctl; *n* = 3). **g** MASMCs were transfected with pLVX-circErbB4, anti-miR-29a-5p, or both (***p < 0.001; ****p < 0.0001; *n* = 3). **h** MASMCs were infected with si-Con or si-Klf-5 + si-IRF-1 + si-circErbB4 for 24 h and then treated with or without AT1-AA for an additional 12 h, and the AT_2_R expression level was detected by Western blot analysis (****p < 0.0001; *n* = 3).

The circErbB4/miR-29a-5p interaction was predicted with Arraystar’s in-house miRNA target prediction software based on TargetScan and miRanda. The results showed that circErbB4 contained sequences complementary to the miR-29a-5p seed sequence (Fig. 7a). The AT1-AA significantly reduced the miR-29a-5p level (Supplementary Fig. S4c). The localization of miR-29a-5p was shown by RNA in situ hybridization in MASMCs after knocking down circErbB4 expression (Fig. 7b). A luciferase assay revealed that an miR-29a-5p-mimic significantly decreased luciferase activity by regulating the wild-type circErbB4 sequence but not a mutant sequence (Fig. 7c). Next, we used biotinylated circErbB4 to pull down miRNA(s) complementary to the circErbB4 sequences. The results revealed that miR-29a-5p was enriched in the circErbB4-pulldown precipitates (Fig. 7d). Consistently, circErbB4 was dramatically enriched in the miR-29a-5p-pulldown precipitates (Supplementary Fig. S4d). In vivo, the circErbB4 level was increased, while the miR-29a-5p level was decreased in the arteries of AT1-AA-treated mice (Supplementary Fig. S4e, f). These findings suggest that circErbB4 may serve as a binding platform for miR-29a-5p.

**Fig. 8:**
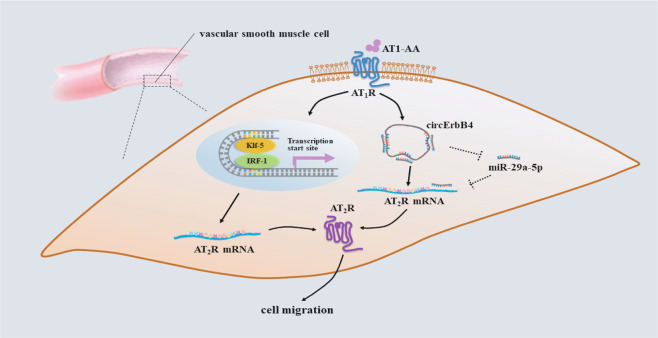
A model of AT_2_R expression level regulation by the AT1-AA-induced Klf-5/IRF-1 and circErbB4/miR-29a-5p axes is shown. AT_2_R expression is regulated through Klf-5/IRF-1 at transcriptional level and circErBb4/miR-29a-5p at posttranscriptional level by AT1-AA.

AT_2_R (Agtr2) was regarded as an miRNA target based on the results from the network (Fig. 6g). We used a bioinformatic approach to find that the mouse AT_2_R 3′-UTR contained two miR-29a-5p-binding sites at nucleotides (Supplementary Fig. S4g). The luciferase assay results showed that the miR-29a-5p-mimic decreased luciferase activity by 50% while the mutation of the miR-29a-5p-binding site completely restored the luciferase activity (Fig. 7e). The miR-29a-5p-mimic and anti-miR-29a-5p (Supplementary Fig. S4h) reduced or increased AT_2_R protein expression, respectively (Fig. 7f). These findings indicated that miR-29a-5p inhibited AT_2_R expression in MASMCs by targeting the AT_2_R 3′-UTR. Furthermore, we found that circErbB4 overexpression plus miR-29a-5p silencing cooperatively upregulated AT_2_R expression (Fig. 7g) and circErbB4 expression knockdown plus treatment with the miR-29a-5p-mimic decreased the AT_2_R expression level (Supplementary Fig. S4i). These data suggested that AT1-AA mediated posttranscriptional regulation of AT_2_R expression in MASMCs via the circErbB4/miR-29a-5p axis. To verify that AT1-AA regulated AT_2_R expression through both IRF-1/Klf-5 and circErbB4/miR-29a-5p, we showed that AT1-AA did not reverse the inhibitory effect of si-IRF-1/Klf-5+si-circErbB4 on the AT_2_R protein level (Fig. 7h). In conclusion, AT1-AA regulated AT_2_R through the following two pathways, i.e., Klf-5/IRF-1-mediated regulation at the transcriptional level and circErbB4/miR-29a-5p-mediated regulation at the posttranscriptional level, and AT1-AA played important roles in regulating the biological function of VSMC migration by increasing AT_2_R expression (Fig. 8).

## Discussion

AT1-AA is known to play a causative role in vascular remodeling through AT_1_R^38,39^. Several reports have confirmed the cross-talk between AT_1_R and AT_2_R^40^. However, the functional role of AT_2_R in the pathological effect of AT1-AA is not completely understood. In our study, we first found that AT1-AA induced aortic remodeling through the induction of VSMC migration while alteration of AT_2_R expression or blocking of AT_2_R signaling with PD123319 reversed these phenomena. The expression of AT_2_R is often upregulated in pathological states associated with cardiovascular diseases^20,41,42^. In this study, we found that AT1-AA increased the expression of AT_2_R but not AT_1_R and promoted cell migration through cytoskeletal reorganization. In contrast, Ang II increased the expression of AT_1_R but not AT_2_R and promoted cell migration through proliferation. These results may explain the different molecular mechanisms involving AT1-AA and Ang II in vascular remodeling. Although the effect of AT1-AA on VSMC migration is better than Ang II, it does not mean that AT1-AA has a more important role in vascular remodeling. Because vascular remodeling is a complex process involving various pathological changes besides migration, such as proliferation, differentiation, apoptosis, inflammation, etc.^43^. We can compare the effects of AT1-AA and Ang II on vascular remodeling in animal model in the further research work. In addition, we found AT1-AA had no significant impact on VSMC proliferation by Brdu assay and supplemental detection of PCNA protein expression (Supplementary Fig. S5), which was opposite to a previous study that AT1-AA promoted VSMC proliferation like Ang II did^11^. It may be due to the same stimulus had different results for the same type but different species^30,44,45^.

As reported previously, AT_2_R expression gradually decreases from fetal life to adulthood^15^. However, the AT_2_R level significantly increases under pathological conditions^41^, and there are some reports supporting that AT_2_R mimics the function of AT_1_R^46^. In our research, we demonstrated the effects of increased AT_2_R expression on VSMC migration and aortic remodeling caused by AT1-AA. An AT1-AA-positive mouse model was constructed in vivo, and the aortic arteries were collected. AT_2_R antagonist treatment attenuated AT1-AA-induced aortic remodeling but failed to reverse high BP. Therefore, we propose that the upregulation of AT_2_R expression may be an early event in vascular remodeling and that the mechanism underlying this upregulation is worth exploring to provide new approaches for preventing vascular lesions.

Since AT_2_R expression was upregulated and played an important role in AT1-AA-induced VSMC migration, we are very interested in the associated molecular mechanism. In our study, we first found that the interaction of IRF-1 with Klf-5 resulted in a synergistic enhancement in AT_2_R expression under AT1-AA treatment. Considering that many genes are transactivated by more than a transcription factor, we speculated that the promotive effect of IRF-1 on AT_2_R might involve other copromoters. Krüppel-like factor (Klf) family proteins are important transcription factors that regulate gene expression in cardiovascular diseases^47^. Our previous study found that Klf-4 regulated the AT_1_R gene and that the function of Klf-5 was opposite that of Klf-4^30,48^; therefore, we speculated that Klf-5 might modulate the AT_2_R gene. The report showed that Klf-4 inhibited IRF-3 bound to the interferon gene promoter^49^. Our results show that Klf-5 can function as a coactivator of IRF-1 in regulating AT_2_R expression because Klf-5 integrates with its binding sites in the AT_2_R promoter.

Furthermore, AT_2_R expression induced by the AT1-AA was regulated not only by transcription factors but also by a circRNA at the posttranscriptional level. Among the top five upregulated circRNAs induced by AT1-AA, we chose the fourth circErbB4, because it was the only one that increased AT_2_R protein level (Supplementary Fig. S6a−d). To provide additional confirmation that the AT1-AA induces circErbB4 formation, we detected the effects on the RNA-binding protein QKI and RNA-editing enzyme ADAR1, which influence circRNA expression. A report shows that knockdown of ADAR1 significantly upregulated circRNA expression; however, ADAR1 antagonizes circRNAs expression on certain conditions^35^. In this study, AT1-AA decreased ADAR1 expression, and we used si-ADAR1 to simulate this decline and investigate the expression of circErBb4. The results showed that knocking down ADAR1 expression had no significant effect on circErbB4 expression, indicating that ADAR1 did not participate in AT1-AA-induced circErbB4 formation. However, knocking down QKI expression significantly decreased circErbB4 formation, and this finding is consistent with previous results showing that QKI regulates circRNAs^50^. Interestingly, we also detected that the AT1-AA decreased the level of miR-29a-5p, which suppressed QKI expression by targeting QKI 3′-UTR (Supplementary Fig. S7a−c). Consistent with our results, miR-29a inhibits QKI-6 expression by binding to the QKI-6 3′-UTR^51^. These data indicated that QKI specifically increased the expression level of circErbB4, thus forming a positive feedback loop between circErbB4 and QKI.

At present, the blocking measures targeting AT1-AA mainly include adsorbing antibodies or blocking receptors^52^. The former approach is under development and currently difficult to apply in the clinic, and the latter strategy mainly includes AT_1_R blockers (ARBs). Some researchers believe that ARBs can block the downstream signaling of AT_1_R and upregulate the expression of AT_2_R to counteract the effect of AT_1_R^53^. However, our current study and reports by others have shown that in some pathological conditions, the upregulation of AT_2_R expression does not necessarily act against AT_1_R and AT_2_R instead acts like AT_1_R, which worsens the severity of the condition. Additionally, ARBs are harmful to pregnant women and fetuses^54^. Therefore, ARBs are not always suitable for blocking AT1-AA and safer and more effective AT1-AA blocking measures must be developed. Our research suggests that AT_2_R may be a new target for intervention.

## Supplementary information

Supplementary Figure S1

Supplementary Figure S2

Supplementary Figure S3

Supplementary Figure S4

Supplementary Figure S5

Supplementary Figure S6

Supplementary Figure S7

supplemental Material
