# Supplementary figures and images for: Increased AT2R expression is induced by AT1R autoantibody via two axes, Klf-5/IRF-1 and circErbB4/miR-29a-5p, to promote VSMC migration
Source: Cell Death Dis. 2020 Jun 8;11(6):432. doi: 10.1038/s41419-020-2643-5 (PMC7280191; doi:10.1038/s41419-020-2643-5)

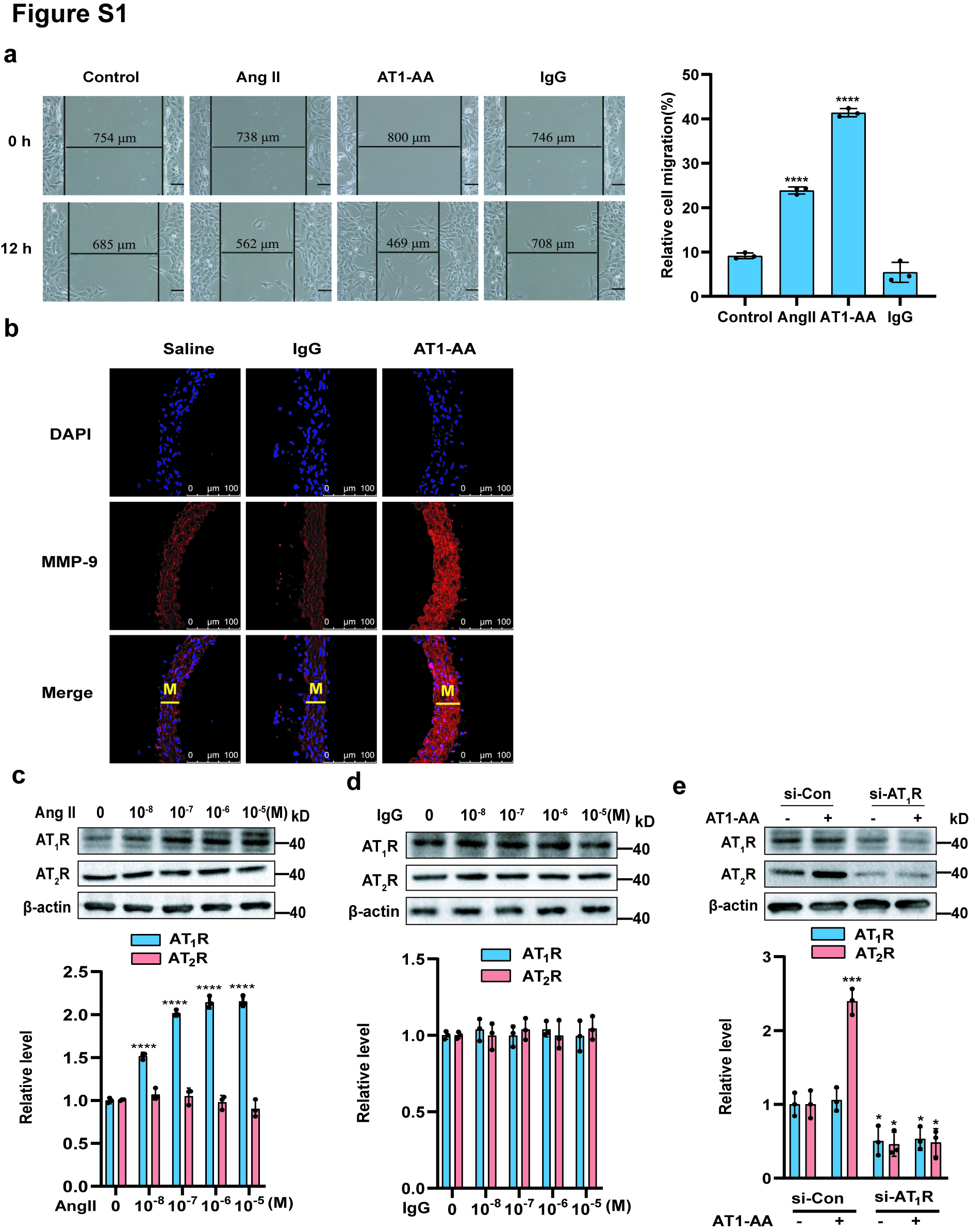

Supplement: Supplementary file 1 — Supplementary Figure S1 [file 41419_2020_2643_MOESM1_ESM.tif]

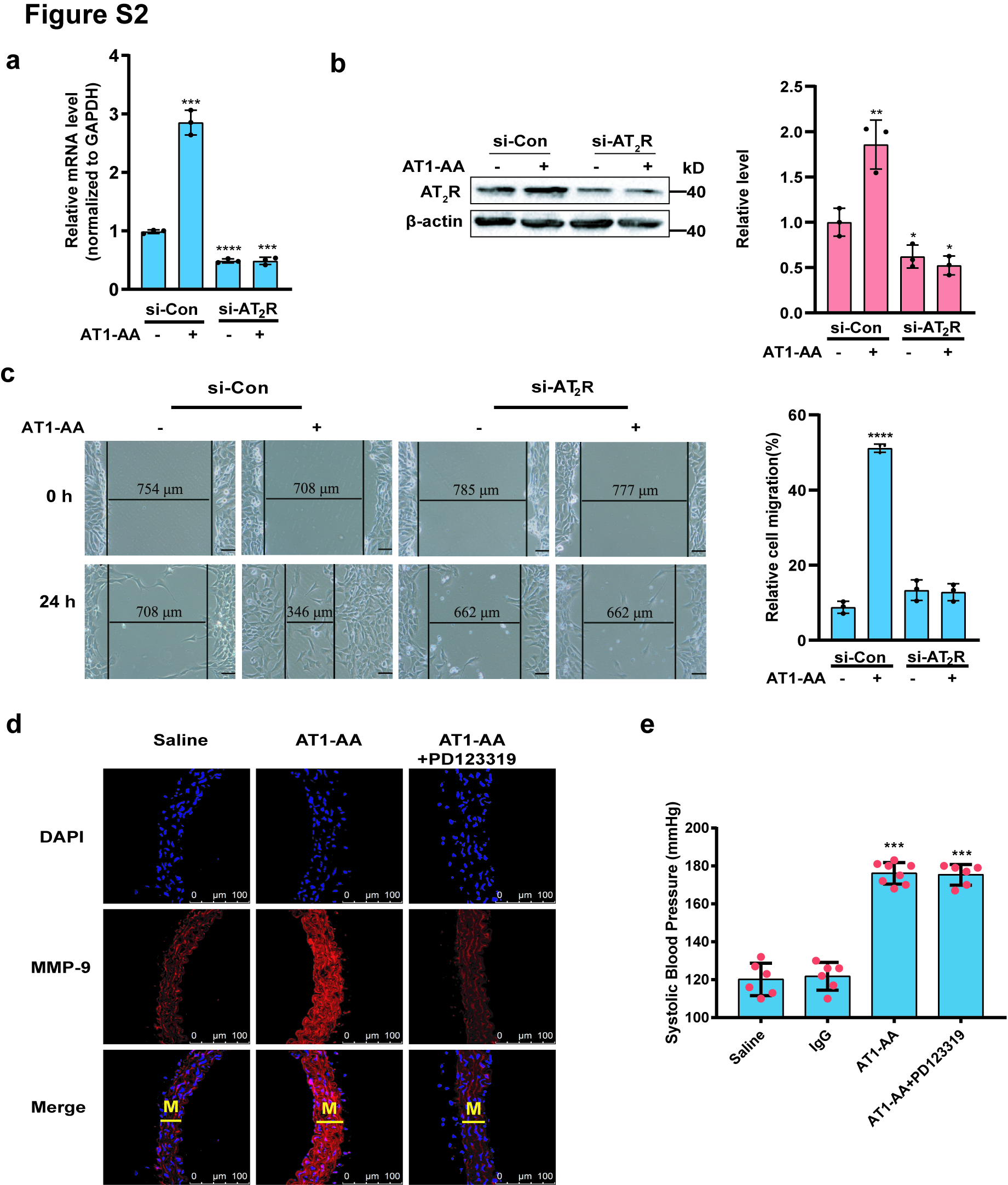

Supplement: Supplementary file 2 — Supplementary Figure S2 [file 41419_2020_2643_MOESM2_ESM.tif]

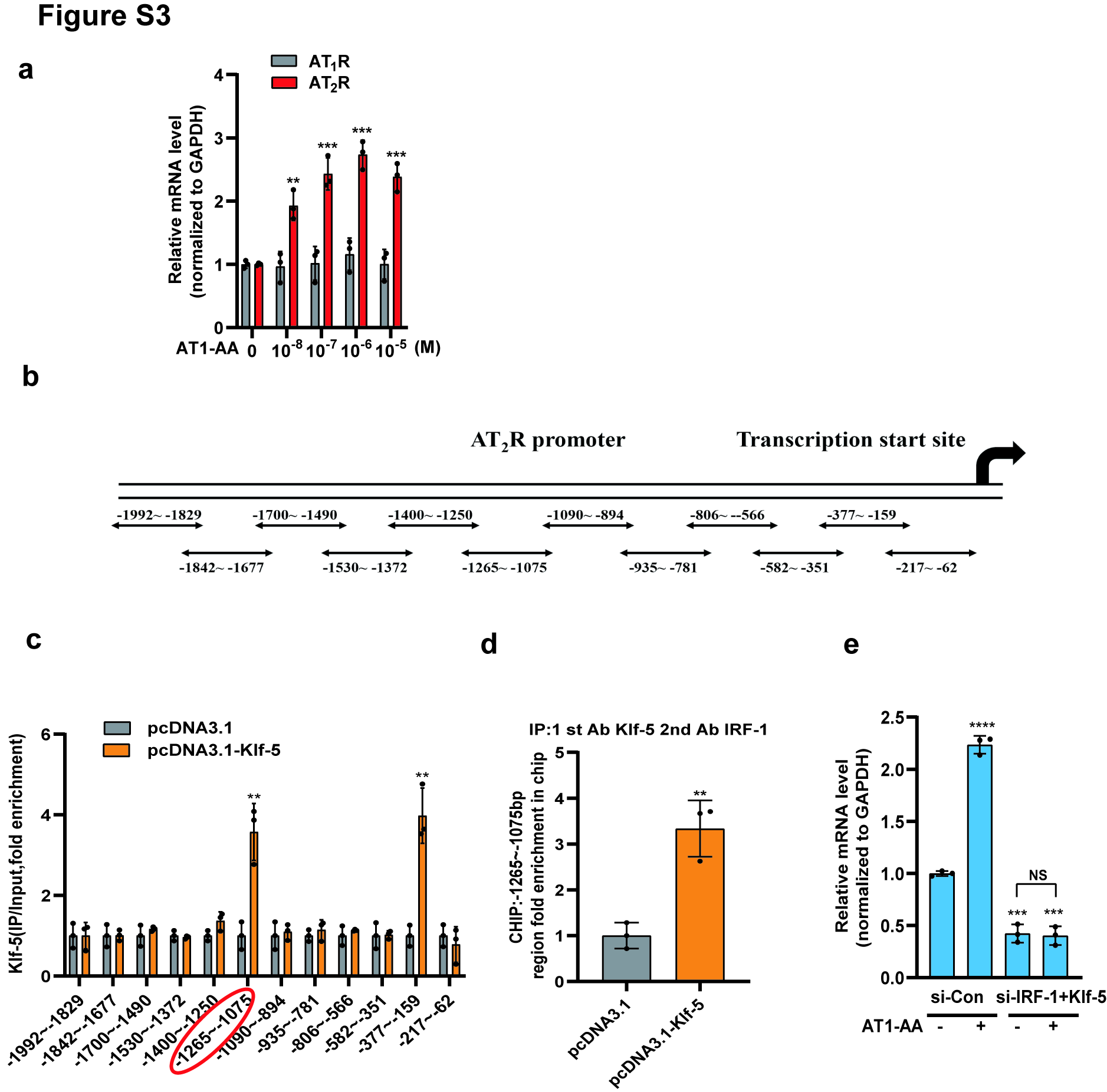

Supplement: Supplementary file 3 — Supplementary Figure S3 [file 41419_2020_2643_MOESM3_ESM.tif]

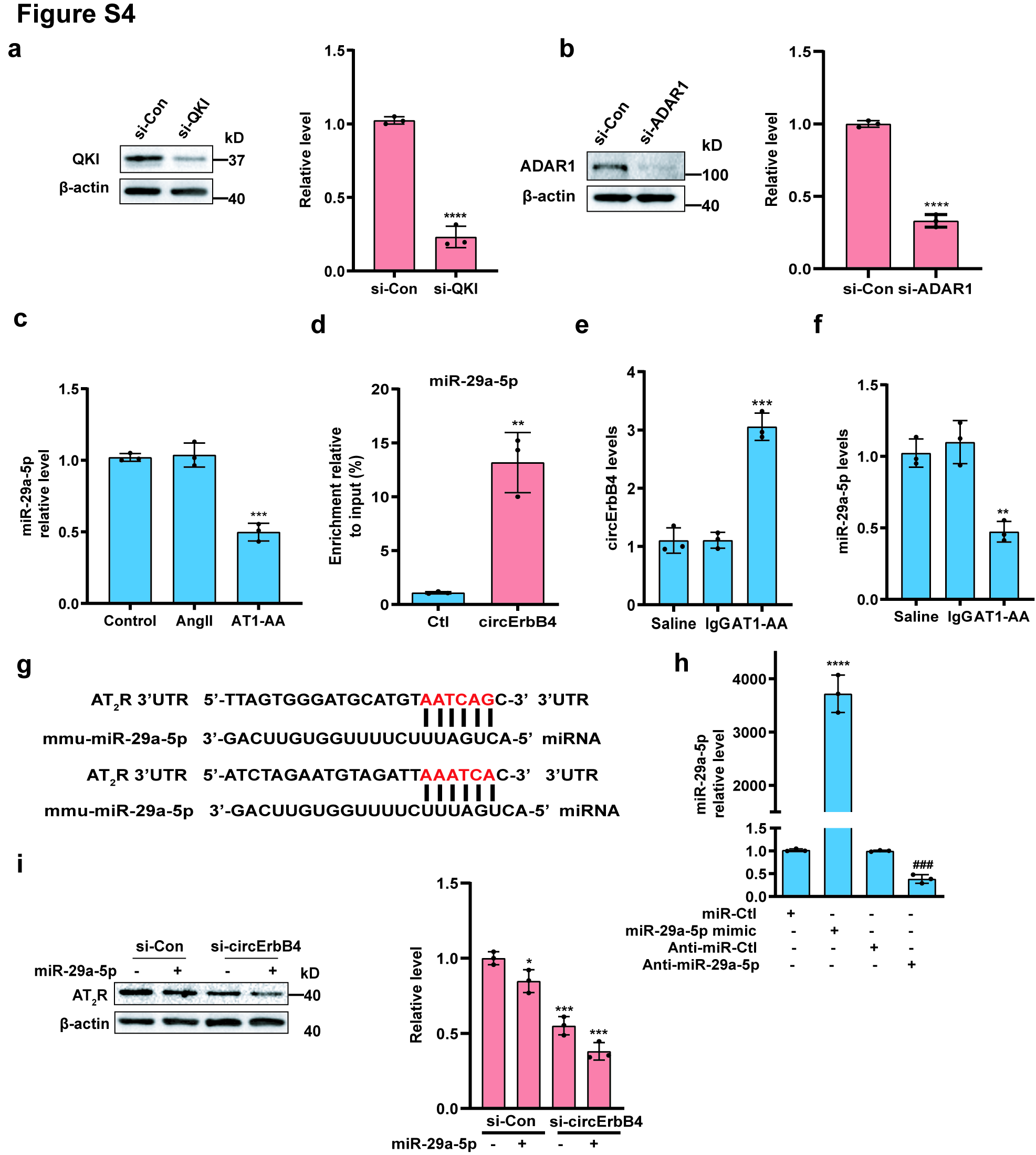

Supplement: Supplementary file 4 — Supplementary Figure S4 [file 41419_2020_2643_MOESM4_ESM.tif]

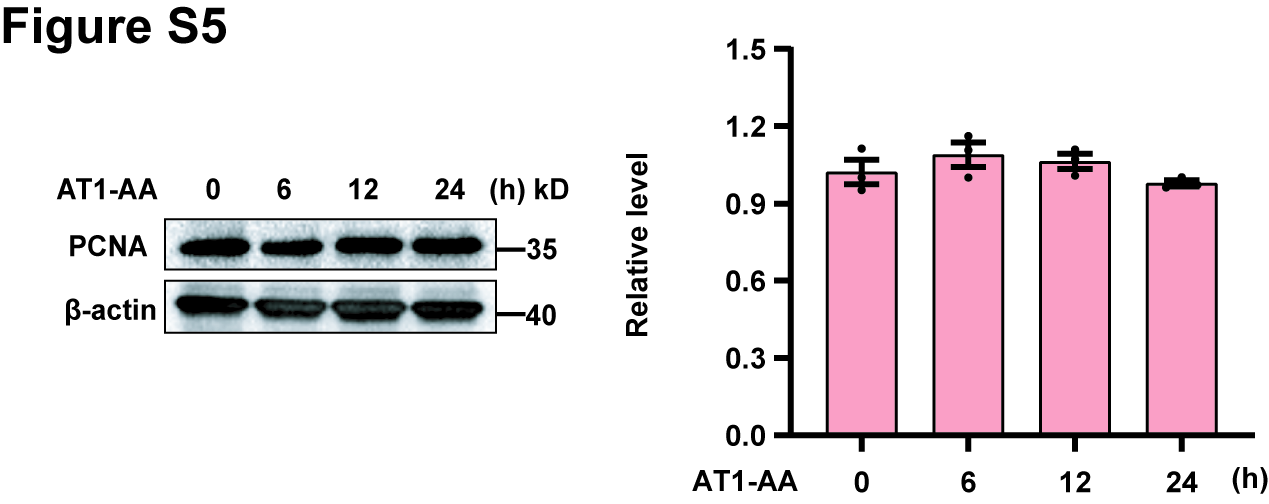

Supplement: Supplementary file 5 — Supplementary Figure S5 [file 41419_2020_2643_MOESM5_ESM.tif]

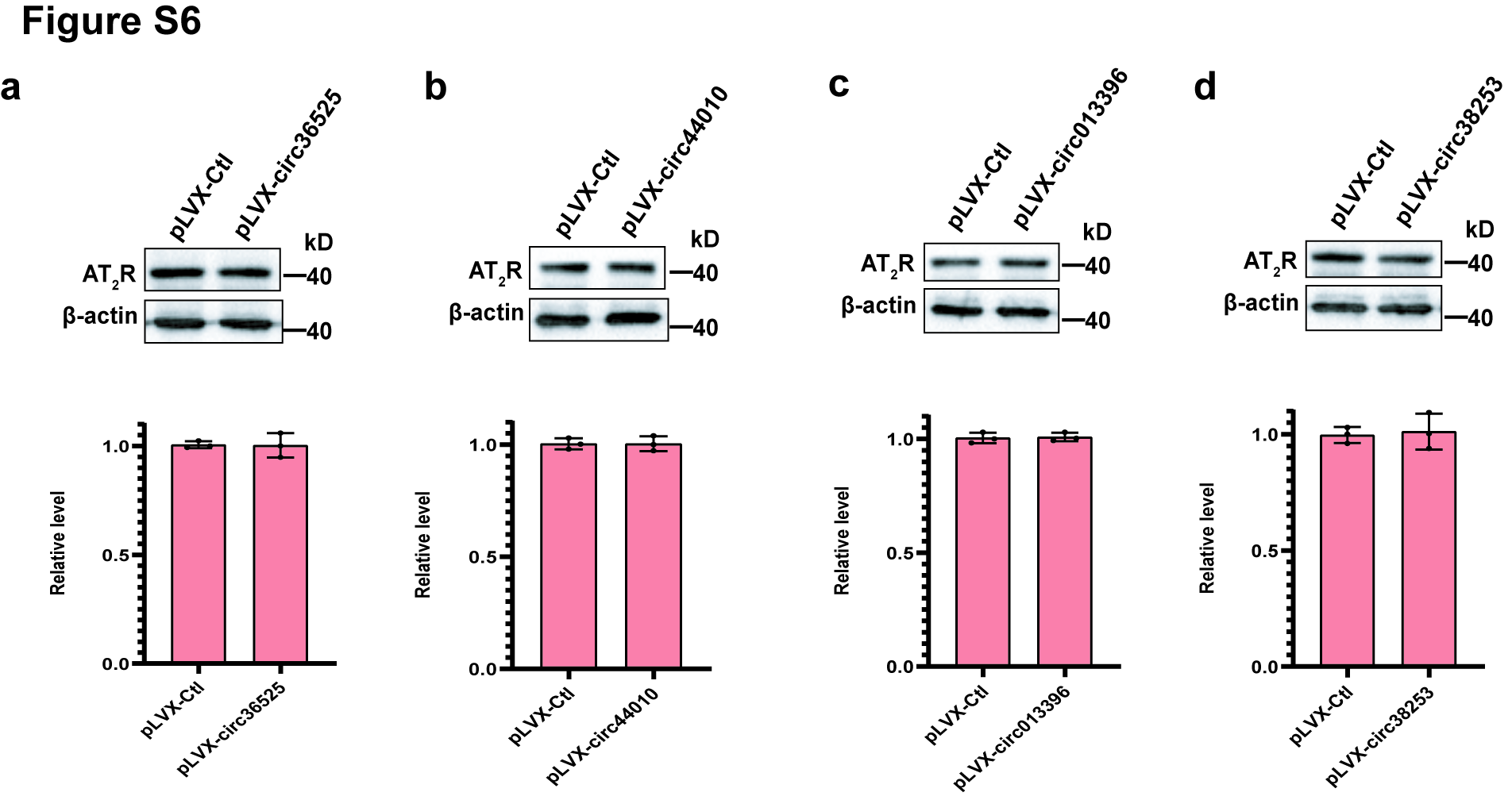

Supplement: Supplementary file 6 — Supplementary Figure S6 [file 41419_2020_2643_MOESM6_ESM.tif]

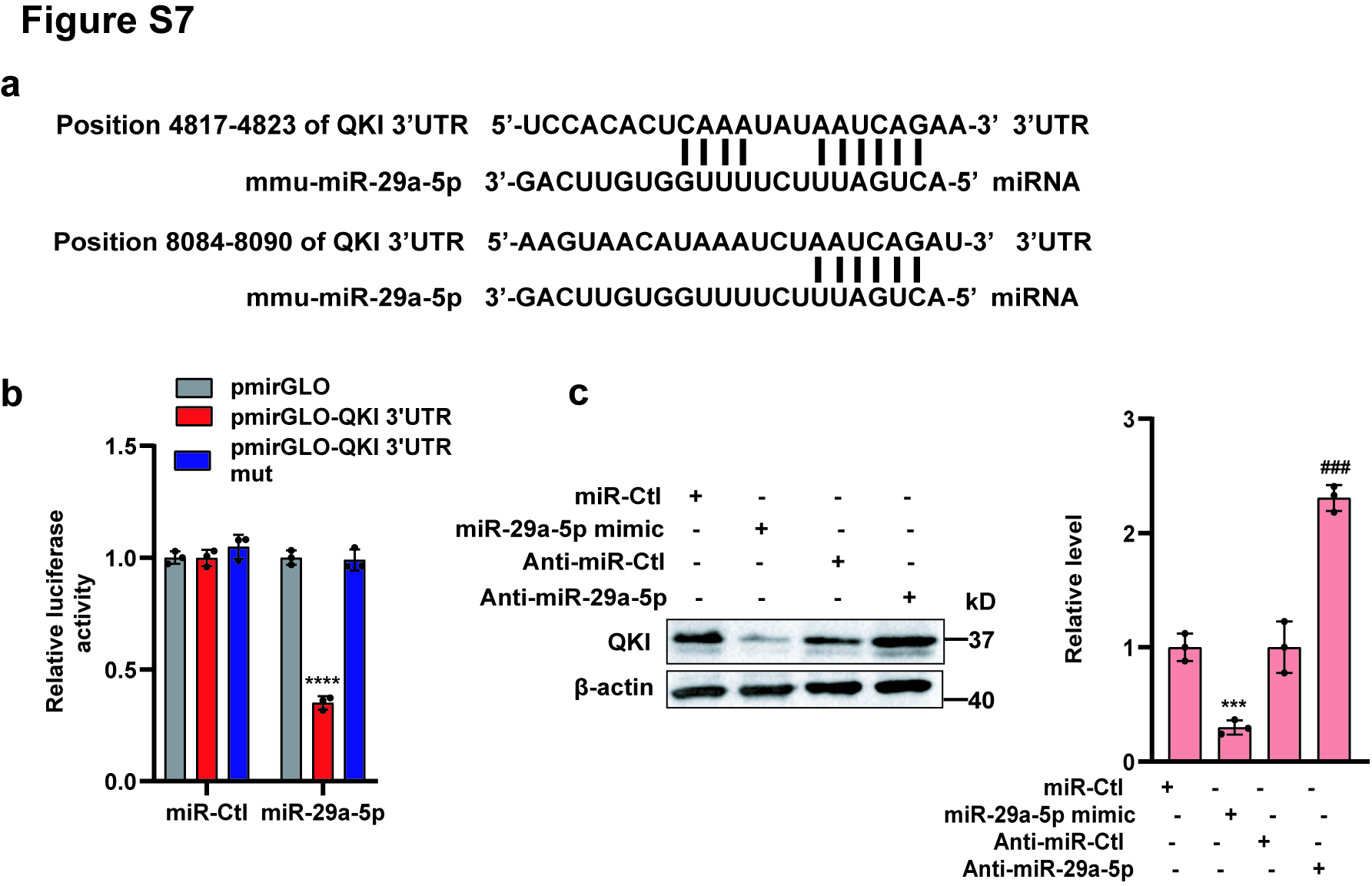

Supplement: Supplementary file 7 — Supplementary Figure S7 [file 41419_2020_2643_MOESM7_ESM.tif]
