# Supplementary material for: Increased AT2R expression is induced by AT1R autoantibody via two axes, Klf-5/IRF-1 and circErbB4/miR-29a-5p, to promote VSMC migration
Source: Cell Death Dis. 2020 Jun 8;11(6):432. doi: 10.1038/s41419-020-2643-5 (PMC7280191; doi:10.1038/s41419-020-2643-5)
Supplement: Supplementary file 8 — supplemental Material [file 41419_2020_2643_MOESM8_ESM.docx]

| **Supplementary Table 1** |  |
| --- | --- |
| List of oligonucleotide sequences | 5'>3' |
| mAT_1_R-F | ACCGCTATGGAATACCGCTG |
| mAT_1_R-R | GAGACACGTGAGCAGGAACA |
| mAT_2_R-F | TACCCGTGACCAAGTCCTGA |
| mAT_2_R-R | TACCCATCCAGGTCAGAGCA |
| mGAPDH-F | AAGGTGAAGGTCGGAGTC |
| mGAPDH-R | GATTTTGGAGGGATCTCG |
| mADAR1-F | CTTGCCGGCACTATGTCTCA |
| mADAR1-R | TCCTAGGGTAAGACTCCGGC |
| mQKI-F | TGAGCAGATGATGTTTTTATGAGAG |
| mQKI-R | ACTCAAAGCATCCGTTTTGGC |
| mmu-circErbB4-divergent-F | GGACAACACTCTTCAGCACCAT |
| mmu-circErbB4-divergent-R | AACGAAACTGGTTGAGGGCC |
| mmu-circErbB4-convergent-F | TGCTCAGGACCAAAGGACAC |
| mmu-circErbB4-convergent-R | TAGAACTAGGGCAGGCTCGT |
| CHIP-(-217-62)-F | AACACATTTGTGGAAACGTTATTAT |
| CHIP-(-217-62)-R | AGAATTCTGG AGCTGACTTATGAAC |
| CHIP-(-377-159)-F | AAAAGATGCTTGTGAACAAAGC |
| CHIP-(-377-159)-R | TAACAGCTCA TTCAAATAAA TCTCAAA |
| CHIP-(-582-351)-F | ACATGAAAAAAAAAAGGCACTAGAT |
| CHIP-(-582-351)-R | AGCAGGCTTT GTTCACAAGC |
| CHIP-(-806-566)-F | AGCTGGTACTGTACAATGCCATC |
| CHIP-(-806-566)-R | CCTTTTTTTT TTCATGTGAC TGTC |
| CHIP-(-935-781)-F | GAGATGCGGCAGAAATTAGAT |
| CHIP-(-935-781)-R | GGTGATGGCA TTGTACAGTA CC |
| CHIP-(-1090-894)-F | TTTTAAGGGCATTTGCTGTG |
| CHIP-(-1090-894)-R | TGGCCTCTGA AGGGCTT |
| CHIP-(-1265-1075)-F | AATGGTGAGACAAAAGAATCAGCT |
| CHIP-(-1265-1075)-R | GCAAATGCCC TTAAAAACTA TTAA |
| CHIP-(-1400-1250)-F | GCTAATACATTTAAGAACACTTTCCC |
| CHIP-(-1400-1250)-R | CTTTTGTCTC ACCATTCTCT GAG |
| CHIP-(-1530-1372)-F | CCCTCTTTCCAGGATCCAT |
| CHIP-(-1530-1372)-R | CAAGGGAAAG TGTTCTTAAA TGTATT |
| CHIP-(-1700-1490)-F | TGCTGCTTCTTGAGACATTTGTA |
| CHIP-(-1700-1490)-R | GTGTGCAATT ACAAGGATTC CC |
| CHIP-(-1842-1677)-F | CAAGTGCTACAATGGTGTCCAC |
| CHIP-(-1842-1677)-R | TTACAAATGT CTCAAGAAGC AGC |
| CHIP-(-1992-1829)-F | TTTCCTAGACATCAGGTTTCCATTA |
| CHIP-(-1992-1829)-R | CATTGTAGCA CTTGTTTTGA TTAAAT |

**Supplementary Figure S1**. **AT1-AA induces VSMC migration and affects AT_2_R expression levels through AT_1_R.** **a** The migratory ability of MASMCs in response to different stimuli was examined by a wound-healing assay. Scale bars=100 μm. Data are presented as mean ± SD (****p<0.0001; n=3). **b** Fluorescent staining of aortic section with an anti-MMP-9 antibody and DAPI was amplified (n=3). **c** and **d** MASMCs were incubated in serum-free medium for 24 h, followed by treatment with Ang II or IgG at the indicated doses. Western blot analysis detected AT_1_R and AT_2_R expression levels (****p<0.0001; n=3). **e** MASMCs were transfected with an siRNA targeting AT_1_R (si-AT_1_R) for 24 h and then treated with or without AT1-AA for an additional 12 h, the expression levels of AT_1_R and AT_2_R were detected by Western blotting (*p<0.05; ***p<0.001; n=3).

**Supplementary Figure S2. AT_2_R participates in AT1-AA-induced VSMC migration.** **a** and **b** MASMCs were transfected with an siRNA targeting AT_2_R (si-AT_2_R) for 24 h and then treated with or without AT1-AA for an additional 12 h, and AT_2_R mRNA was detected by qRT-PCR. Data are presented as mean ± SD (***p<0.001; ****p<0.0001; n=3). Western blotting was performed using anti-AT_2_R and anti-β-actin antibodies (*p<0.05; **p<0.01; n=3). **c** MASMCs were transfected with si-Con or si-AT_2_R, treated with or without the AT1-AA and stained for a wound-healing assay. Scale bars=100 μm (****p<0.0001, n=3). **d** Fluorescent staining performed detected MMP-9 expression after 1 month in saline-treated mice, AT1-AA (with or without PD123319)-treated mice. AT1-AA+PD123319 group was compared with saline and AT1-AA groups in Supplementary Figure S1b. **e** Systolic arterial blood pressure was measured. Data represent the mean ± SD (***p<0.001, n=6-8).

**Supplementary Figure S3. Klf-5 and IRF-1 promote the transcription of the AT_2_R gene.** **a** Relative mRNA levels of AT_1_R and AT_2_R were examined by qRT-PCR and are presented after normalizing to the GAPDH level. Data are presented as mean ± SD (**p<0.01; ***p<0.001; n=3). **b** A schematic representation of the promoter of AT_2_R is shown, and primers for different regions of the AT_2_R promoter were used for amplification. **c** ChIP analysis was used to evaluate the Klf-5 occupancy at the AT_2_R promoter (**p<0.01; n=3). **d** A two-step ChIP assay identified the interaction between Klf-5 and IRF-1 (**p <0.01; n=3). **e** MASMCs were infected with si-Con or si-Klf-5+si-IRF-1 for 24 h and then treated with or without AT1-AA for an additional 12 h. AT_2_R mRNA was detected by qRT-PCR (***p<0.001; ****p<0.0001; n=3).

**Supplementary Figure S4. AT1-AA regulates AT_2_R expression through the circErbB4/miR-29a-5p axis.** **a** and **b** MASMCs were transfected with an siRNA targeting QKI (si-QKI) or ADAR1 (si-ADAR1) for 24 h. Western blotting was performed using anti-QKI, anti-ADAR1, and anti-β-actin antibodies. Data are presented as mean ± SD (****p<0.0001; n=3). **c** The miR-29a-5p expression level was examined by qRT-PCR (***p<0.001; n=3). **d** qRT-PCR was used to detect miR-29a-5p enriched from MASMC lysates with a biotinylated oligonucleotide probe for circErbB4 (**p<0.001; n=3). **e** and **f** The expression levels of circErbB4 and miR-29a-5p in arteries from the saline, IgG and AT1-AA groups were examined by qRT-PCR (**p<0.01; ***p<0.001 *vs.* the saline group; n=3). **g** The miR-29a-5p-binding site in the 3’-UTR of the AT_2_R mRNA transcript is shown in red. **h** qRT-PCR was used to detect miR-29a-5p levels in MASMCs transfected with a miR-29a-5p mimic, anti-miR-29a-5p or the corresponding control. Data represent the mean ± SD of three independent experiments (****p<0.0001 *vs.* miR-Ctl; ###p<0.001 *vs.* anti-miR-Ctl). **i** MASMCs were transfected with si-circErbB4, miR-29a-5p, or both. AT_2_R expression was analyzed by Western blotting (*p<0.05; ***p<0.001; n=3).

**Supplementary Figure S5. AT1-AA has no impact on PCNA expression.** PCNA protein level was analyzed by Western blotting. The right panel shows densitometric analyses from three independent experiments. Data are presented as mean ± SD.

**Supplementary Figure S6. Other circRNAs can’t upregulate AT_2_R level. a-d** The expression level of AT_2_R in MASMCs transfected with pLVX-circ36525, pLVX-circ44010, pLVX-circ013396 or pLVX-circ38253 was assessed by Western blotting.

**Supplementary Figure S7. miR-29a-5p suppresses QKI expression by targeting the QKI 3’-UTR. a** The miR-29a-5p-binding site in the 3’-UTR of the QKI mRNA transcript was amplified. **b** HEK 293A cells were cotransfected with a miR-29a-5p mimic and the wild-type pmirGLO-QKI 3’-UTR plasmid or mutant pmirGLO-QKI 3’-UTR mut plasmid. After 24 h, luciferase activities were measured. (****p<0.001 *vs.* pmirGLO or pmirGLO-QKI 3’-UTR mut; n=3). **c** MASMCs were transfected with the miR-29a-5p mimic, anti-miR-29a-5p, or the corresponding control. QKI expression levels were analyzed by Western blotting. The right panel shows densitometric analyses from three independent experiments (***p<0.001 *vs.* miR-Ctl; ###p<0.001 *vs.* Anti-miR-Ctl).
